# How do traditional masculinity ideologies and emotional competence relate to aggression and physical domestic violence in cisgender men?

**DOI:** 10.3389/fpsyg.2023.1100114

**Published:** 2023-03-14

**Authors:** Flora Logoz, Lukas Eggenberger, Nikola Komlenac, Michèle Schneeberger, Ulrike Ehlert, Andreas Walther

**Affiliations:** ^1^Department of Clinical Psychology and Psychotherapy, Psychological Institute, University of Zurich, Zürich, Switzerland; ^2^Institute of Diversity in Medicine, Medical University of Innsbruck, Innsbruck, Tyrol, Austria

**Keywords:** traditional masculinity ideology, emotional competence, domestic violence, aggression, conformity to masculinity ideology

## Abstract

**Background:**

Men are disproportionately often perpetrators of physical domestic violence (DV). Gender role constructs, such as traditional masculinity ideologies (TMI), are broadly accepted as an explanation for this effect. Emotional competence further constitutes an important role in TMI and the prevention of DV. However, the interactions between these constructs remains unclear.

**Objective:**

The present study aims to investigate associations of TMI with aggression, DV perpetration, and emotional competence, while also examining emotional competence as a potential moderator.

**Method:**

A sample of 428 cisgender men (*M*_age_ = 43.9 ± 15.3) from German-speaking countries in Europe completed an anonymous online survey that assessed TMI, aggression, and DV perpetration as well as alexithymia, emotion regulation, and self-compassion as indicators of emotional competence.

**Results:**

Strong TMI were associated with high levels of aggression and overall reduced emotional competence, as reflected by high levels of alexithymia, frequent use of expressive suppression, and low levels of self-compassion. Strong conformity to TMI was associated with a higher likelihood for DV perpetration when considering relevant sociodemographic covariates. Moderation analyses revealed that expressive suppression buffered the association between TMI and DV perpetration.

**Conclusion:**

Men with strong TMI report high levels of aggression and impaired emotional competence. While strong conformity to TMI was associated with more frequent perpetration of DV, higher expressive suppression seems to buffer the association between TMI and DV perpetration. The present study highlights the importance of addressing gender ideologies when working on aggression, DV perpetration and emotional competence in men.

## 1. Introduction

Violence is considered a major public health concern (Krug et al., 2002; World Health Organization, 2021). A large percentage of violent acts are committed in the context of domestic violence (DV), which can be defined as the exertion of power, including assaultive and coercive behaviors, to manipulate, control, and dominate a partner or a child living in the same household as the perpetrator (Hornor, 2005). DV includes all forms of physical, sexual, psychological, or economic violence (Howard et al., 2010). Violence by intimate partners or family members affects women and children disproportionately often, with global estimates suggesting that about a third of women experience lifetime violence from an intimate partner (World Health Organization, 2005, 2021; Oram et al., 2017) and over 50% of children worldwide have experienced past-year DV (Hillis et al., 2016). Women and children affected by DV are at an increased risk for severe short- and long-term consequences, such as physical injuries, impaired mental health, increased psychosomatic symptoms, increased need of healthcare, and even fatal outcomes (Campbell et al., 2002; Hornor, 2005; World Health Organization, 2005, 2016; Flury et al., 2010). Affected children are further at risk of achieving lower educational outcomes (Romano et al., 2015), increased delinquency (Doelman et al., 2021), and repeating DV during adulthood (Siegel, 2013). Given the high prevalence and far-reaching consequences of physical DV, identifying key factors facilitating DV against women and children is paramount, especially because addressing these risk factors can directly contribute to the prevention of DV (Guedes et al., 2016). Thus, the present study aims to contribute to the understanding and identification of risk factors for gender-based violence and further advance the prevention of physical DV.

To date, the greatest risk factor to engage in injurious and severe DV is being male, as the vast majority of violent offenders and severe DV perpetrators are men (Anderson, 1997; Kimmel, 2002; World Health Organization, 2021). While biological determinism falls short of providing an adequate explanation for gender-based violence in human society (Archer et al., 2005; Pavlov et al., 2012; Walther et al., 2017, 2019), social role theories are broadly accepted as a rationale for gender differences in the display of aggression and DV perpetration (Ali and Naylor, 2013; McQuigg, 2017; Walther et al., 2021). Among social role theories, second-wave feminist scholarship highlights the upholding of inequity through gender ideologies as a basis for the social construction of gender (being masculine) in contrast to biological sex (being male; Connell, 2005; Connell and Messerschmidt, 2005). Gender ideologies, such as traditional masculinity ideologies (TMI) describe a socially defined set of standards and norms of how men and women are traditionally expected to be and behave in society (Pleck, 1995; Levant, 1996; Levant and Richmond, 2016). Therein, men but not women are expected to be in control of many (social) situations and even use physical force (i.e., be aggressive or violent) to put through their own interests (Levant and Richmond, 2016).

Interpersonal processes of social reinforcement and observational learning are thought to promote conformity to gender-aligned behavior. Thus, TMI inform boys and men about socially reinforced and gender-aligned ways to experience and express emotions (Levant, 2011; Levant and Richmond, 2016). The primary negative emotion that men are socially permitted and encouraged to exhibit is anger (Fields and Cochran, 2011), which, in turn, increases the accessibility of aggressive scripts among men. Thus, social role theories suggest that higher endorsement of and conformity to TMI is associated with increased display of anger and aggression, which are conductive of DV perpetration. Indeed, various studies have reported associations between endorsement of TMI and self-reported aggression (Thomas and Levant, 2012), perpetration of violence against intimate partners (Reidy et al., 2014), or with problematic attitudes and behaviors toward women and marginalized groups (Levant and Richmond, 2008; O’Neil, 2013; Gerdes et al., 2018).

However, DV may not only be associated with TMI, but also closely associated with emotional competence. While TMI is related to difficulties in processing and expressing emotions (e.g., alexithymia), and emotionally dysregulated behavior (Lease et al., 2010; Levant et al., 2014; Levant and Richmond, 2016), emotional competence includes the ability to functionally recognize, express, and regulate emotions (McRae and Gross, 2020). Anger management courses for male DV perpetrators, for example, focus on emotional competence dimensions, such as increasing empathy formation and perspective taking to compensate for potential developmental deficits in emotional competence (Pickover, 2010). Examining men participating in batter intervention programs revealed that endorsement of TMI and emotional competence emerged as the most relevant factors explaining 25% of the variance in violence perpetration against intimate partners (Tager et al., 2010). The authors further concluded that men who exhibit strong endorsement of TMI and experience high levels of negative and overwhelming affect due to an insufficient emotion regulation capacity are particularly likely to perpetrate DV. However, no interaction between TMI and emotional competence on their potential to explain IPV was investigated.

Thus, it is highly interesting that low emotional competence skills – a crucial protective factor against the general use of violence – shows a strong negative association with the endorsement of TMI (Levant et al., 2009a; Reilly et al., 2014). For example, endorsement of TMI has been directly related to impaired emotion processing, emotional inexpressiveness, and lower interpersonal competencies (Lease et al., 2010; Levant et al., 2014; Levant and Richmond, 2016). Thus, in conjunction with emotional competence, TMI could account for gender differences in aggression as a form of emotionally dysregulated behavior (Cole et al., 1996; Bohnert et al., 2003). This presumption is further supported by a recent study by Berke et al. (2019). Therein, the link between masculine discrepancy stress (i.e., men’s finding themselves not living up to TMI) and DV perpetration was found to be mediated by difficulties in emotion regulation.

Further evidence of the association between TMI and impaired emotional competence can be found in the Normative Male Alexithymia Hypothesis (Levant, 1992). Therein, mild-to-moderate levels of alexithymia (i.e., difficulties recognizing, understanding, naming, and expressing emotions; Neimah et al., 1976; Krystal, 1982) among men are understood to be a result of masculine gender role socialization. Namely, socialization in a culture where TMI are prevalent being inherently traumatic due to the harsher sanctioning of gender non-conform behaviors in boys and men (e.g., McCreary, 1994; Levy et al., 1995; Sirin et al., 2004) leads to higher levels of alexithymia in men, particularly among those strongly endorsing TMI. However, a recent study by Levant et al. (2014) suggests that the traumatic impact of TMI may have been overestimated. Levant et al. (2014) conclude that alexithymia may be better explained by emotion regulation, such as emotion suppression, rather than processes related to trauma.

Emotion regulation serves to successfully adapt to one’s environment by modifying emotional responding (Gross, 1999). Considering the downregulation of negative emotional states, the two most prominently investigated emotion regulation strategies are cognitive reappraisal and expressive suppression (Gross, 1999, 2002). While reappraisal is considered a functional strategy by allowing individuals to successfully reduce negative affect, expressive suppression is more often associated with negative consequences, such as is increased physiological arousal, and often elicits a rebound effect that makes the suppressed emotion even more prominent (Gross, 2002; Haga et al., 2009; Ehring et al., 2010). Gender differences in emotion regulation, particularly men showing consistently higher levels of expressive suppression, further point to an association between gender-based ideologies, such as TMI, and emotion regulation (Haga et al., 2009). Consequently, prominent measures of TMI include respective subdimensions that reflect attitudes in favor of expressive suppression (Gerdes et al., 2018), such as restrictive emotionality (e.g., “A men should never admit when others hurt his feelings”; Levant et al., 2013) or emotional control (e.g., the reverse-scored item “I tend to share my feelings”; Levant et al., 2020). However, it is important to note that these subdimensions are conceptually distinct from emotion regulation. This is easily demonstrated for restrictive emotionality because it reflects an attitude rather than an emotion regulation strategy. Similarly, despite including a behavioral component, emotional control is conceptually different from expressive suppression because it does not determine through which emotion regulation strategies it is attained.

Another functional form of emotional competence, which has become increasingly important in resilience and well-being research, is self-compassion (Neff et al., 2007). Self-compassion entails three dimensions: Self-kindness (a forgiving attitude toward oneself versus self-criticism), common humanity (understanding struggles as human condition versus isolation), and mindfulness (Neff et al., 2007). Thus, instead of suppressing their negative emotions, self-compassionate individuals accept their struggle as part of the human condition and treat themselves with kindness. TMI, however, seems incompatible with self-compassion, as ideals like restrictive emotionality (e.g., “Men should be detached in emotionally charged situations”; Levant et al., 2013) and toughness (e.g., “It is important for a man go take risks, even if he might get hurt”; Levant et al., 2013) stand in conflict with self-kindness and promote self-criticism instead. Concordantly, Reilly et al. (2014) found that higher levels of self-compassion were associated with reduced adherence to traditional masculine norms, providing further empirical support for the strong conceptual contrast between self-compassion and TMI.

Taken together, processes of social learning encourage gender-specific responses to different emotions (Berke et al., 2018). Through social rewards for restrictive emotionality, socialization under TMI may thus impair the development of emotional competence in men. In reverse, emotional competence in men with high TMI may buffer against aggression and physical DV. Thus, in a first step, the present study aims to replicate the positive relationship between TMI, aggression, and physical DV, as well as the negative relationship between TMI and emotional competence. In a second step, the present study further aims to explore possible interaction effects of key emotional competence dimensions on the association between TMI, aggression, and physical DV. Namely, we propose the following hypotheses:

(1) Strong TMI are associated with higher levels of aggression and more frequent perpetration of DV.
(2) Strong TMI are associated with impaired emotional competence (i.e., rare cognitive reappraisal, frequent expressive suppression, high levels of alexithymia, and low self-compassion).
(3) High emotional competence (i.e., frequent cognitive reappraisal, rare expressive suppression, low levels of alexithymia, and high self-compassion) has a buffering effect on the association between TMI and DV perpetration.

## 2. Methods

### 2.1. Study design

**Figure 1:**
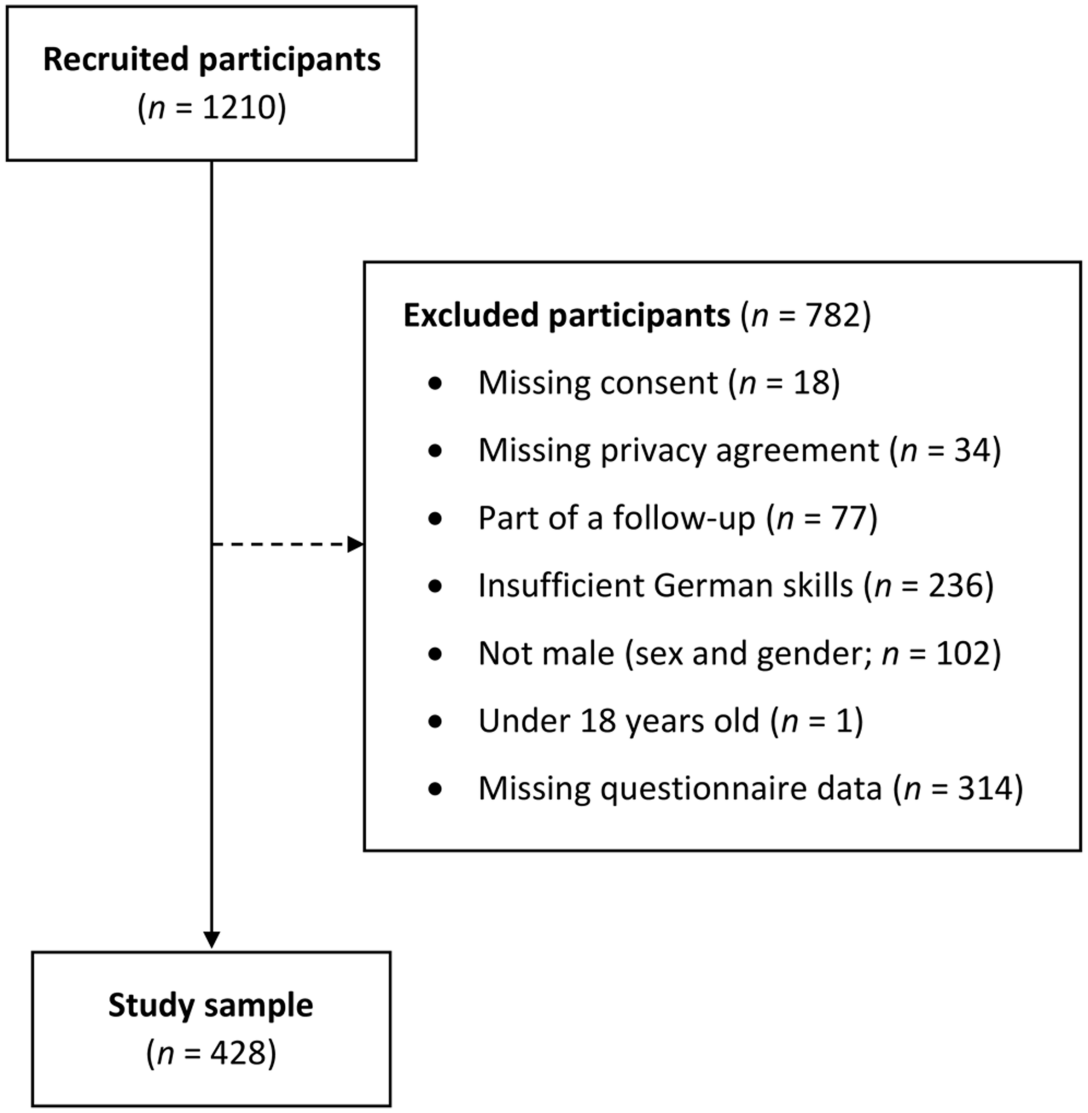
Overview of the exclusion process. *n* = Number of participants.

The present pre-registered cross-sectional study used data from an anonymous online survey called *Andromind Self-Test* (AST), accessible *via* the website *andromind.ch*, which informs men about gender role norms and men-specific mental health issues. Participation was based on self-selection as the subjects were recruited through advertisement on social media. Only participants providing informed consent and accepting the data privacy agreement could proceed to the survey. Subsequent inclusion criteria consisted of age (being 18 years or older), sufficient German skills to complete the online questionnaire, and being male while also identifying with one’s birth-assigned gender. Participants who did not provide complete data for the examined constructs were excluded from the analysis. A detailed overview of the exclusion process is presented in Figure 1. Prior to data collection, the study was registered to the *Open Science Framework* (OSF) and approved by the University of Zurich Ethics Commission.

### 2.2. Instruments

#### 2.2.1. Sociodemographic information

Participants’ sociodemographic information was assessed, of which a detailed overview can be found in the Supplementary (Supplementary Table S1). Participants were asked separately about their gender identity and sex assigned at birth. Subsequent questions asked participants’ age, nationality, highest completed education, sexual orientation, marital status, and intimate and exclusive relationship status. Additionally, current psychiatric diagnosis, psychological burden, and psychotherapy use was assessed by self-report. For some of the analyses, individual subgroups were aggregated due to their small size (e.g., gay, bisexual, asexual, and other sexuality were aggregated to non-heterosexual).

#### 2.2.2. Male role norms inventory – Short form

The Male Role Norms Inventory – Short Form (MRNI-SF; Levant et al., 2013) assesses endorsement of TMI (i.e., whether participants believe that men should be or beave in accordance with TMI) on seven subscales with three items each: *Restrictive Emotionality*, *Self-Reliance Trough Mechanical Skills*, *Negativity Toward Sexual Minorities*, *Avoidance of Femininity*, *Importance of Sex*, *Dominance*, and *Toughness*. Participants are asked their agreement with different statements about TMI (e.g., “A man should never admit when others hurt his feelings”) using a 7-point Likert scale (1 = *strongly disagree to* 7 = *strongly agree*). For the English version, Cronbach’s α ranges from 0.79 to 0.90 with *α* = 0.92 for the total score. The German version of the MRNI-SF has adequate validity and reliability (Komlenac and Hochleitner, 2022), with α between 0.73 and 0.89 for individual subscales. In the present study, a total scale score was used (i.e., higher scores indicating stronger endorsement of TMI) which possessed a reliability in the present sample of *α* = 0.94 and McDonald’s *ω* = 0.95.

#### 2.2.3. Conformity to masculine norms inventory – 30

The Conformity to Masculine Norms Inventory – 30 (CMNI-30; Levant et al., 2020) consists of 30 items that assess the level of conformity to masculine norms (e.g., “The women in my life should obey me”) with the use of a 6-point Likert scale (0 = *strongly disagree* to 5 = *strongly agree*). The CMNI-30 comprises 10 underlying dimensions of conformity to TMI, namely *Emotional Control*, *Winning*, *Playboy*, *Violence*, *Heterosexual Self-Presentation*, *Pursuit of Status*, *Primacy of Work*, *Power over Women*, *Self-Reliance*, and *Risk-Taking*. For the original English version internal consistencies ranging from *α* = 0.72 to *α* = 0.94 have been reported (Levant et al., 2020). The German translation was reported to have internal consistencies of *ω* = 0.63 to *ω* = 0.94 and to be invariant across sexual orientation, i.e., measuring the same constructs with the same accuracy in heterosexually identified and sexual minority men (Komlenac et al., 2023). Furthermore, for heterosexually identified cisgender men the validation of the German version of the CMNI-30 supported the use of a total score for TMI. Therefore, in the present sample, the total scale score was used, with higher scores indicating stronger conformity to TMI, showed a reliability of *α* = 0.85 and *ω* = 0.88.

#### 2.2.4. Toronto alexithymia scale – 26

The Toronto Alexithymia Scale – 26 (TAS-26; Taylor et al., 1985) contains 26 items that measure the degree of alexithymia (e.g., “It is difficult for me to find the right words for my feelings”) on four subscales: *Difficulty Describing Feelings*, *Difficulty Identifying Feelings*, *Externally Oriented Thinking*, and *Reduced Daydreaming*. Participants are asked the extent to which they agree with each statement using a 5-point Likert scale (1 = *strongly disagree*, 5 = *strongly agree*). Thus, higher scores indicate higher levels of alexithymia. The current study used a slightly shorter German-language version consisting of only 18-items (excluding the *Reduced Daydreaming* subscale and two items with high cross-factor-loadings), which showed the highest reliability and validity in a representative German population sample (*α* = 0.81; Kupfer et al., 2000). In the present sample, the total score of the TAS-26 showed a reliability of *α* = 0.83 and *ω* = 0.88.

#### 2.2.5. Emotion regulation questionnaire – 10

The Emotion Regulation Questionnaire (ERQ; Gross and John, 2003) measures the frequency of use of cognitive reappraisal (e.g., “I control my emotions by changing the way I think about the situation I’m in”) and expressive suppression (e.g., “I keep my emotions to myself”) on a 7-point Likert scale (1 = *not true at all* to 7 = *very true*). Higher scores mean more frequent use of cognitive reappraisal and expressive suppression, respectively. For the German translation, Abler and Kessler (2009) reported adequate internal consistencies and convergent validity, with *α* = 0.74 (suppression) and *α* = 0.76 (reappraisal). In the present sample, the reappraisal subscale possessed a reliability of *α* = 0.73 and *ω* = 0.83, and the suppression subscale showed a reliability of *α* = 0.63 to *ω* = 0.70.

#### 2.2.6. Self-compassion scale – D – Short form

The Self-Compassion Scale – D – Short Form (SCS-D-SF; Hupfeld and Ruffieux, 2011) is the German translation of the Self-Compassion Scale – Short Form (SCS-SF; Raes et al., 2011). Both consist of 12 items assessing self-compassion on six dimensions: S*elf-Kindness*, *Self-Judgement*, *Common Humanity, Isolation*, *Mindfulness*, and *Over-Identification*. Participants are asked how often they behave in a certain manner (e.g., “When something painful happens I try to take a balanced view of the situation”) with possible responses on a 5-point Likert scale (1 = *almost never* to 5 = *almost always*). Adequate internal consistency has been reported for both the English version with *α* ≥ 0.86 (Raes et al., 2011) and the German translation with *α* = 0.84 (Hupfeld and Ruffieux, 2011), as well as strong correlations with the original Self-Compassion Scale (SCS; Raes et al., 2011). In the present study, a total scale score was used, where higher values indicate more self-compassion, and which possessed a reliability of *α* = 0.89 and *ω* =0.91.

#### 2.2.7. Buss-Perry aggression questionnaire

The Buss and Perry Aggression Questionnaire (BPAQ; Buss and Perry, 1992; Herzberg, 2003) measures general aggression with 29 items and four subscales, namely *Physical Aggression*, *Verbal Aggression*, *Anger*, and *Hostility*. Participants are asked, how characteristic a given description is of themselves (e.g., “Given enough provocation, I may hit another person”) using a 5-point Likert scale (0 = *extremely uncharacteristic of me* to 4 = *extremely characteristic of me*). A psychometric evaluation of the German translation confirms support for validity and reliability with α ranging from 0.75 to 0.88 (Herzberg, 2003). In the current study, a total score was used, where higher values indicate higher levels of aggression, with a reliability of *α* = 0.91 and *ω* = 0.92.

#### 2.2.8. Marlowe-Crowne social desirability scale

A short form of the Marlowe-Crowne Social Desirability Scale (MC-SDS; Crowne and Marlowe, 1960; Vésteinsdóttir et al., 2017) with 10 items was used to assess participant’s social desirability their response style. Participants have to answer whether they would ascribe socially desirable traits or behaviors to themselves (e.g., “I have never intensely disliked anyone”) using dichotomous response style (0 = *No*, 1 = *Yes*). Thus, higher scores indicate greater social desirability in participant’s response style. The short form of the English version possessed a composite reliability of ρx = 0.83 (Vésteinsdóttir et al., 2017). Because no German-language version has been validated so far, a forward-translated German version was used in the present study, which showed a reliability of *α* = 0.61 and *ω* = 0.67.

#### 2.2.9. Physical domestic violence

Physical DV was assessed in five questions described in the following, using a dichotomous *yes*-*no* answer format. Participants were first asked if they had felt a higher potential of violence toward their partner and/or children within the past month. Then, two questions asked about perpetration of physical DV within the past month (“During the last month, have you ever been physically violent towards your partner or children?”) and within lifetime (“Have you ever been physically violent towards your partner or children?”). Furthermore, to include as a control variable in the analyses participants were asked about their experience of physical DV, again for the past-month (“During the last month, have you ever experienced physical violence from your partner or children?”) and lifetime (“Have you ever experienced physical violence from your partner or children?”). For all questions, physical violence was specified as “including behaviors such as hitting someone with a fist or flat hand, kicking someone with the feet, strong painful holding/grabbing/squeezing of someone, choking someone by the neck, pulling someone’s hair, or hitting someone with objects such as a belt.”

The self-constructed item assessing one of the main outcome variables of the present study, namely lifetime perpetration of physical DV showed an agreement rate of 84.2% over a four- to eight-week period among 57 participants who completed the survey twice. Convergent validity was further determined by a positive point-biserial correlation between lifetime perpetration of DV and the *Physical Aggression* scale of the BPAQ (*r_pb_* = 0.21, *p* < 0.001, *n* = 628).

### 2.3. Statistical analyses

First, descriptive statistics were calculated, and psychometric properties of the study questionnaires were assessed by their internal consistencies (Cronbach’s α; McDonald’s ω) and distribution of their raw scores (graphically and by skewness and kurtosis; Supplementary Table S2; Supplementary Figure S1). Pearson’s correlation coefficient was then calculated for the assessment of bivariate associations among all questionnaires and lifetime perpetration of physical DV.

The analysis for the main research question consisted of two parts. In a first part, linear (OLS) and binomial logistic (MLE) regression analyses were conducted with TMI (MRNI-SF; CMNI-30) as individual predictor variables and emotional competencies (TAS-26; ERQ-reappraisal; ERQ-suppression; SCS-12), aggression (ABPQ), and lifetime perpetration of physical DV as individual outcome variables. Each predictor – outcome combination was analyzed with three sequential regression models, including the following steps: (1) no covariates, (2) including participant’s age, education, sexual orientation, relationship status, and social desirability (MC-SDS) as covariates, and (3) including participants’ lifetime experience of physical DV as an additional covariate.

In a second part, a potential moderating effect of emotional competencies on the association between TMI, aggression, and perpetration of physical DV was examined by introducing an interaction term (TMI × emotional competence) into the respective model, irrespective of whether significant main effects were present. As in the first part, each predictor – outcome combination was analyzed with three sequential regression models, (1) no covariates, (2) including sociodemographic covariates, and (3) including lifetime experienced DV.

The assumptions for robust statistical inference in regression analyses were assessed through visual inspection of the residuals, as well as through quantitative evaluation of Cook’s distances for highly influential points (Cook, 1977) and the generalized variance inflation factor for collinearities (Fox and Monette, 1992). Furthermore, standardized regression coefficients were obtained with the Agresti method (Menard, 2011). For all analyses, an alpha level of 0.05 was used to test for statistical significance, subsequently applying a familywise Holm-Bonferroni correction for multiple testing (Holm, 1979). All calculations were performed in the *R* software environment (version 4.1.2; R Core Team, 2020) including Wickham’s (2016) package *ggplot2* for visualizations and Revelle’s (2020) package *psych* for the estimation of bivariate correlations, psychometric properties of the questionnaires, and the implementation of the Holm-Bonferroni procedure.

## 3. Results

### 3.1. Participants

As presented in Table 1, the study sample consisted of 428 cisgender men with a mean age of 43.9 years (SD = 15.3). The majority of men was of German nationality (69.6%), finished either a secondary (49.3%) or tertiary (45.1%) education, self-identified as heterosexual (77.3%). A larger number of participants was unmarried (54.2%) than married (32.7%), and about as many men were currently in an exclusive intimate partnership (46.3%) as were not in an intimate partnership (47.0%). Furthermore, about one-third self-reported to currently be formally diagnosed with a psychiatric disorder (30.8%), more than half stated to currently be psychologically burdened (53.5%), and one-fifth were currently using psychotherapy (20.3%). Regarding perpetration and experience of physical DV, 59 men (13.8%) reported having used DV against their partner or children at some point in their lives, and 70 men (16.4%) reported having experienced physical DV from their partner or children during the course of their life.

**Table 1:** Descriptive statistics of the sample (*n* = 428).

Age, mean (SD)	43.9 (15.3)
Nationality, *n* (%)	
Swiss	92 (21.5)
German	298 (69.6)
Austrian	28 (6.5)
Luxembourg	1 (0.2)
Other	9 (2.1)
Education, *n* (%)	
None completed	1 (0.2)
Secondary education	211 (49.3)
Tertiary education	193 (45.1)
Other	23 (5.4)
Sexual Orientation, *n* (%)	
Heterosexual	331 (77.3)
Gay	53 (12.4)
Bisexual	32 (7.5)
Asexual	1 (0.2)
Other	3 (0.7)
Not sure/no answer	8 (1.9)
Marital Status, *n* (%)	
Unmarried	232 (54.2)
Married/registered partnership	145 (33.9)
Separated	51 (11.9)
Intimate Relationship, *n* (%)	
Yes	198 (46.3)
Yes, non-exclusive	29 (6.8)
No	201 (47.0)
Current Mental Health, *n* (%)	
Psychiatric diagnosis	132 (30.8)
Psychological burden	229 (53.5)
Psychotherapy use	87 (20.3)
Domestic Violence, *n* (%)	
Increased potential (past month)	16 (3.7)
Used (past month)	5 (1.2)
Used (lifetime)	59 (13.8)
Experienced (past month)	8 (1.9)
Experienced (lifetime)	70 (16.4)

*n*, Number of participants; SD, Standard deviation.

### 3.2. Descriptive statistics

For each questionnaire, the following item-level mean scores were found: endorsement of TMI 2.2 (SD = 1.0, range = 1; 6.6), conformity to TMI 1.8 (SD = 0.6, range = [0.4, 4.5]), alexithymia 2.5 (SD = 0.6, range = [1, 4.4]), cognitive reappraisal 4.2 (SD = 1.1, range = [1, 7]), expressive suppression 3.9 (SD = 1.0, range = [1, 7]), self-compassion 3.0 (SD = 0.8, range = [1.2, 4.9]), aggression 1.4 (SD = 0.6, range = [0.2, 3.3]), and social desirability 0.4 (SD = 0.2, range = [0, 1]). Participant-level mean scores of each questionnaire are reported in Supplementary Table S2.

### 3.3. Correlational analysis

As presented in Table 2, strong endorsement of TMI was associated with higher conformity to TMI, increased expressive suppression, increased aggression, and lower self-compassion. No associations were found between endorsement of TMI and alexithymia or cognitive reappraisal. Strong conformity to TMI, on the other hand, was linked with increased alexithymia, increased expressive suppression, more aggression, and lower self-compassion. No association between conformity to TMI and cognitive reappraisal was found. Further associations were found between high aggression and frequent perpetration of DV, increased alexithymia, increased expressive suppression, lower cognitive reappraisal, and lower self-compassion. Additional analyses using the individual CMNI-30 subscales are provided in the supplementary materials (Supplementary Table S3).

**Table 2:** Pearson’s correlation coefficients of the questionnaires.

	1.	2.	3.	4.1	4.2	5.	6.	7.
1. MRNI-SF	–							
2. CMNI-30	0.58^***^	–						
3. TAS-26	0.06	0.25^***^	–					
4.1 ERQ-reappraisal	0.02	−0.01	−0.25^***^	–				
4.2 ERQ-suppression	0.20^***^	0.34^***^	0.44^***^	0.31^***^	–			
5. SCS-SF	−0.16^*^	−0.36^***^	−0.60^***^	0.41^***^	−0.20^***^	–		
6. BPAQ	0.35^***^	0.48^***^	0.31^***^	−0.22^***^	0.15^*^	−0.51^***^	–	
7. MC-SDS	−0.14	−0.37^***^	−0.17^**^	0.11	−0.04	0.34^***^	−0.51^***^	–
8. DV perpetration	0.10	0.08	0.04	−0.04	0.01	−0.03	0.21^***^	−0.11

*p*-values were adjusted for multiple testing using the Holm-method. MRNI-SF, Male Role Norms Inventory – Short Form; CMNI-30, Conformity to Masculine Norms Inventory – 30; TAS-26, Toronto Alexithymia Scale – 26; ERQ, Emotion Regulation Questionnaire; SCS-SF, Self-Compassion Scale – Short Form; BPAQ, Buss-Perry Aggression Questionnaire; MC-SDS, Marlowe-Crowne Social Desirability Scale; DV, domestic violence (0 = no perpetration, 1 = perpetration). Bold formatting for statistical significance on an alpha-level of 5%. ^*^p < 0.05; ^**^p < 0.01; ^***^p < 0.001.

### 3.4. Multiple regression analysis

As presented in Table 3, we used linear regression model that were controlled for sociodemographic variables (age, education, sexual orientation, intimate relationship status, and social desirability), experienced physical DV, and corrected for multiple testing, to examine the separate association between both TMI measures and aggression. Results showed that strong endorsement of TMI as well as strong conformity to TMI were both individually associated with increased aggression among men. Additional sensitivity analyses for heterosexual-identified men only (Supplementary Tables S3, S4) showed no differences in heterosexual-identified men as compared to the total sample.

**Table 3:** Linear regression models with aggression (ABPQ) as outcome variable.

Predictor		*β*^a^ (SE)	95% CI	*B* ^raw^	*p*	*p* (corr.)	ΔR^2^ (%)
(A) Endorsement of TMI as predictor							
(1)	MRNI-SF	6.27 (0.81)	[4.68, 7.86]	0.30	**<0.001**	–	
							12.1^***^
(2)	MRNI-SF	5.42 (0.71)	[4.03, 6.81]	0.26	**<0.001**	**<0.001**	
	Age	0.20 (0.74)	[−1.25, 1.65]	0.01	0.790	0.790	
	Education	−4.50 (1.46)	[−7.38, −1.62]	−4.50	**0.002**	**0.009**	
	Sexual orientation	2.80 (1.66)	[−0.46, 6.06]	2.80	0.091	0.183	
	Relationship	−4.16 (1.41)	[−6.92, −1.40]	−4.16	**0.003**	**0.010**	
	MC-SDS	−8.39 (0.70)	[−9.76, −7.01]	−3.78	**<0.001**	**<0.001**	
							25.2^***^
(3)	MRNI-SF	5.28 (0.70)	[3.89, 6.66]	0.25	**<0.001**	**<0.001**	
	Age	0.06 (0.73)	[−1.38, 1.51]	0.00	0.929	0.929	
	Education	−4.54 (1.45)	[−7.40, −1.68]	−4.54	**0.002**	**0.010**	
	Sexual orientation	2.96 (1.65)	[−0.28, 6.20]	2.96	0.073	0.146	
	Relationship	−4.19 (1.40)	[−6.93, −1.44]	−4.19	**0.003**	**0.011**	
	MC-SDS	−8.13 (0.70)	[−9.51, −6.75]	−3.66	**<0.001**	**<0.001**	
	DV experienced	4.84 (1.87)	[1.16, 8.52]	4.84	**0.010**	**0.030**	
							0.8^*^
(B) Conformity to TMI as predictor							
(1)	CMNI-30	8.66 (0.76)	[7.17, 10.14]	0.49	**<0.001**	–	
							23.3^***^
(2)	CMNI-30	6.44 (0.74)	[4.99, 7.89]	0.36	**<0.001**	**<0.001**	
	Age	1.80 (0.73)	[0.36, 3.24]	0.12	**0.015**	**0.029**	
	Education	−4.80 (1.44)	[−7.63, −1.97]	−4.80	**<0.001**	**0.004**	
	Sexual orientation	2.02 (1.61)	[−1.16, 5.19]	2.02	0.212	0.212	
	Relationship	−4.53 (1.38)	[−7.25, −1.82]	−4.53	**0.001**	**0.004**	
	MC-SDS	−6.92 (0.73)	[−8.35, −5.49]	−3.12	**<0.001**	**<0.001**	
							16.1^***^
(3)	CMNI-30	6.34 (0.73)	[4.89, 7.78]	0.36	**<0.001**	**<0.001**	
	Age	1.63 (0.73)	[0.19, 3.06]	0.11	**0.026**	0.053	
	Education	−4.84 (1.43)	[−7.65, −2.04]	−4.84	**<0.001**	**0.004**	
	Sexual orientation	2.22 (1.60)	[−0.93, 5.38]	2.22	0.166	0.166	
	Relationship	−4.55 (1.37)	[−7.24, −1.86]	−4.55	**<0.001**	**0.004**	
	MC-SDS	−6.66 (0.73)	[−8.09, −5.23]	−3.00	**<0.001**	**<0.001**	
	DV experienced	5.18 (1.83)	[1.58, 8.79]	5.18	**0.005**	**0.015**	
							1.0^**^

SE, standard error; corr., adjusted for multiple testing using the Holm-method; ΔR^2^, difference of adjusted R^2^ between consecutive models; MRNI-SF, Male Role Norms Inventory – Short Form; CMNI-30, Conformity to Masculine Norms Inventory – 30; MC-SDS, Marlowe-Crowne Social Desirability Scale. Reference category is non-tertiary education, heterosexual, and single. Bold formatting for significant findings on an alpha-level of 5%. ^a^Standardized coefficient; ^raw^unstandardized coefficient ^*^p < 0.05; ^**^p < 0.01; ^***^p < 0.001.

Binomial logistic regression analyses with lifetime perpetration of DV as an outcome variable (Table 4) revealed that strong endorsement of TMI was linked to frequent use of physical DV. This effect did not remain statistically significant after including sociodemographic variables and experienced physical DV as covariates. Strong conformity to TMI was by itself not associated with lifetime-perpetration of physical DV. This association was significant after including sociodemographic covariates and experienced physical DV but did not survive a correction for multiple testing. Additional sensitivity analyses for heterosexual-identified men only (Supplementary Table S5) revealed no differences when compared to the total sample.

**Table 4:** Binomial logistic regression models with DV perpetration as outcome variable.

Predictor		*β*^a^ (SE)	OR^a^	95% CI	OR^raw^	*p*	*p* (corr.)	ΔR^2^ (%)
(A) Endorsement of TMI as predictor								
(1)	MRNI-SF	0.26 (0.13)	1.30	[1.01, 1.68]	1.01	**0.044**	–	
								1.5^*^
(2)	MRNI-SF	0.20 (0.15)	1.22	[0.91, 1.63]	1.01	0.190	0.379	
	Age	0.95 (0.19)	2.58	[1.79, 3.72]	1.06	**<0.001**	**<0.001**	
	Education	−0.96 (0.33)	0.38	[0.20, 0.72]	0.38	**0.003**	**0.012**	
	Sexual orientation	−0.94 (0.47)	0.39	[0.16, 0.97]	0.39	**0.044**	0.131	
	Relationship	0.04 (0.31)	1.04	[0.56, 1.91]	1.04	0.907	0.907	
	MC-SDS	−0.51 (0.16)	0.60	[0.44, 0.83]	0.80	**0.002**	**0.009**	
								17.4^***^
(3)	MRNI-SF	0.14 (0.16)	1.15	[0.83, 1.58]	1.01	0.392	0.784	
	Age	0.99 (0.20)	2.70	[1.81, 4.02]	1.07	**<0.001**	**<0.001**	
	Education	−1.14 (0.35)	0.32	[0.16, 0.64]	0.32	**0.001**	**0.006**	
	Sexual orientation	−0.91 (0.49)	0.40	[0.15, 1.05]	0.40	0.063	0.190	
	Relationship	0.04 (0.33)	1.04	[0.54, 2.00]	1.04	0.903	0.903	
	MC-SDS	−0.38 (0.17)	0.68	[0.49, 0.95]	0.84	**0.024**	0.095	
	DV experienced	2.05 (0.34)	7.74	[3.94, 15.22]	7.74	**<0.001**	**<0.001**	
								13.0^***^
(B) Conformity to TMI as predictor								
(1)	CMNI-30	0.22 (0.13)	1.25	[0.96, 1.63]	1.01	0.093	–	
								1.2
(2)	CMNI-30	0.34 (0.16)	1.40	[1.01, 1.93]	1.02	**0.042**	0.103	
	Age	1.03 (0.19)	2.81	[1.92, 4.10]	1.07	**<0.001**	**<0.001**	
	Education	−1.01 (0.33)	0.36	[0.19, 0.69]	0.36	**0.002**	**0.011**	
	Sexual orientation	−0.99 (0.47)	0.37	[0.15, 0.93]	0.37	**0.034**	0.103	
	Relationship	0.04 (0.31)	1.04	[0.56, 1.91]	1.04	0.903	0.903	
	MC-SDS	−0.41 (0.17)	0.66	[0.47, 0.92]	0.83	**0.014**	0.056	
								18.8^***^
(3)	CMNI-30	0.37 (0.18)	1.44	[1.00, 2.08]	1.02	**0.048**	0.190	
	Age	1.09 (0.21)	2.96	[1.96, 4.47]	1.07	**<0.001**	**<0.001**	
	Education	−1.20 (0.36)	0.30	[0.15, 0.61]	0.30	**<0.001**	**0.004**	
	Sexual orientation	−0.91 (0.48)	0.40	[0.16, 1.04]	0.40	0.060	0.190	
	Relationship	0.04 (0.33)	1.04	[0.54, 2.00]	1.04	0.907	0.907	
	MC-SDS	−0.29 (0.18)	0.75	[0.53, 1.06]	0.88	0.106	0.212	
	DV experienced	2.08 (0.35)	7.98	[4.04, 15.78]	7.98	**<0.001**	**<0.001**	
								13.2^***^

SE, standard error; OR, odds ratio; corr., adjusted for multiple testing using the Holm-method; ΔR^2^, difference of Nagelkerke’s *R*^2^ between consecutive models; MRNI-SF, Male Role Norms Inventory – Short Form; CMNI-30, Conformity to Masculine Norms Inventory – 30; MC-SDS, Marlowe-Crowne Social Desirability Scale. Reference category is non-tertiary education, heterosexual, and single. Bold formatting for significant findings on an alpha-level of 5%. ^a^standardized coefficient; ^raw^unstandardized coefficient ^*^p < 0.05; ^**^p < 0.01; ^***^p < 0.001.

Additional linear regression analyses examining the association between TMI and emotional competencies, controlled for covariates and multiple testing, revealed that strong endorsement of TMI was linked to frequent expressive suppression (Supplementary Table S6) and reduced self-compassion (Supplementary Table S7). Strong conformity to TMI was associated with high levels of alexithymia (Supplementary Table S8), frequent expressive suppression (Supplementary Table S6), and reduced self-compassion (Supplementary Table S7). No relationship was found between endorsement of TMI nor conformity to TMI and cognitive reappraisal (Supplementary Table S9).

### 3.5. Moderation analysis

In order to examine a potential moderating effect of emotional competencies on the association between TMI and aggression as well as TMI and perpetration of physical DV, separate interaction terms (TMI x emotional competencies) were added to the respective regression models, again controlled for sociodemographic covariates, and corrected for multiple testing. A moderating effect of self-compassion on the association between endorsement of TMI and aggression was found (Supplementary Table S10), where men with high (≥ median; *n* = 221) self-compassion showed a weaker association between endorsement of TMI and aggression (*β* = 3.39, 95% CI [1.61–5.17]) as compared to men with low (< median; *n* = 207) self-compassion (*β* = 7.43, 95% CI [5.22–9.65]). However, this effect did not persist after controlling for covariates and was not present for conformity to TMI as a moderator.

Alexithymia moderated the association between conformity to TMI and perpetration of physical DV (Supplementary Table S11), indicating that among men with high levels (≥ median; *n* = 221) of alexithymia the association between conformity to TMI and perpetration of physical DV (OR = 1.00, 95% CI [0.59–1.68]) was weaker as compared to men with low (< median; *n* = 207) alexithymia (OR = 1.90, 95% CI [1.05–3.45]). The moderating effect did not remain significant after correcting for multiple testing and was not found for endorsement of TMI as a moderator.

As presented in Table 5, expressive suppression emerged as a moderator for the association between endorsement of TMI and perpetration of physical DV as well as conformity to TMI and perpetration of physical DV. Men with high (≥ median; *n* = 216) expressive suppression showed a significantly weaker association between endorsement of TMI and the likelihood of having ever used physical DV (OR = 0.93, 95% CI [0.56–1.56]) as compared to men with low (< median; *n* = 212) expressive suppression (OR = 1.39, 95% CI [0.91–2.11]). Similarly, men with high expressive suppression also showed a significantly weaker association between conformity to TMI and the likelihood of having ever used physical DV (OR = 1.32, 95% CI [0.68–2.56]) as compared to men with low expressive suppression (OR = 1.63, 95% CI [1.05–2.54]). However, the moderating effects did not withstand a correction for multiple testing. Additional sensitivity analyses for these moderation analyses that include heterosexual-identified men only (Supplementary Table S12) showed, again, no substantial differences between heterosexual-identified men only and the total sample.

**Table 5:** Moderation models with DV perpetration as outcome and expressive suppression as moderator.

Predictor		*β*^a^ (SE)	OR^a^	95% CI	OR^raw^	*p*	*p* (corr.)	ΔR^2^ (%)
(A) Endorsement of TMI as predictor								
(1)	MRNI-SF	0.28 (0.14)	1.33	[1.01, 1.74]	1.09	**0.039**	0.078	
	ERQ-suppress.	0.02 (0.15)	1.02	[0.77, 1.37]	1.24	0.865	0.865	
	(MRNI x suppress.)	−0.39 (0.14)	0.67	[0.52, 0.88]	1.00	**0.004**	**0.013**	
								5.4^**^
(2)	MRNI-SF	0.18 (0.15)	1.20	[0.88, 1.62]	1.11	0.242	0.726	
	ERQ-suppress.	0.19 (0.17)	1.21	[0.87, 1.67]	1.39	0.261	0.726	
	(MRNI x suppress.)	−0.53 (0.16)	0.59	[0.43, 0.80]	0.99	**<0.001**	**0.005**	
	Age	1.02 (0.20)	2.77	[1.88, 4.06]	1.07	**<0.001**	**<0.001**	
	Education	−1.00 (0.34)	0.37	[0.19, 0.71]	0.37	**0.003**	**0.015**	
	Sexual orientation	−1.08 (0.49)	0.34	[0.13, 0.88]	0.34	**0.026**	0.104	
	Relationship	0.09 (0.32)	1.09	[0.59, 2.04]	1.09	0.779	0.779	
	MC-SDS	−0.54 (0.17)	0.58	[0.42, 0.80]	0.78	**0.001**	**0.006**	
								18.1^***^
(3)	MRNI-SF	0.11 (0.17)	1.11	[0.80, 1.55]	1.08	0.529	1	
	ERQ-suppress.	0.19 (0.17)	1.21	[0.86, 1.70]	1.31	0.280	0.839	
	(MRNI x suppress.)	−0.42 (0.16)	0.66	[0.48, 0.91]	0.99	**0.010**	0.062	
	Age	1.06 (0.21)	2.88	[1.90, 4.35]	1.07	**<0.001**	**<0.001**	
	Education	−1.16 (0.36)	0.31	[0.15, 0.64]	0.31	**0.001**	**0.010**	
	Sexual orientation	−1.05 (0.50)	0.35	[0.13, 0.94]	0.35	**0.037**	0.149	
	Relationship	0.08 (0.34)	1.09	[0.56, 2.11]	1.09	0.803	1	
	MC-SDS	−0.42 (0.17)	0.65	[0.46, 0.92]	0.83	**0.015**	0.073	
	DV experienced	1.95 (0.35)	7.00	[3.50, 13.98]	7.00	**<0.001**	**<0.001**	
								10.9^***^
(B) Conformity to TMI as Predictor								
(1)	CMNI-30	0.32 (0.15)	1.37	[1.02, 1.85]	1.10	**0.039**	0.078	
	ERQ-suppress.	−0.01 (0.15)	0.99	[0.74, 1.32]	1.31	0.934	0.934	
	(CMNI x suppress.)	−0.37 (0.14)	0.69	[0.53, 0.90]	0.99	**0.006**	**0.018**	
								4.7^*^
(2)	CMNI-30	0.33 (0.18)	1.39	[0.97, 1.99]	1.11	0.075	0.226	
	ERQ-suppress.	0.08 (0.17)	1.08	[0.78, 1.50]	1.38	0.642	1	
	(CMNI x suppress.)	−0.42 (0.16)	0.66	[0.48, 0.89]	0.99	**0.007**	**0.047**	
	Age	1.03 (0.19)	2.82	[1.92, 4.12]	1.07	**<0.001**	**<0.001**	
	Education	−0.91 (0.33)	0.40	[0.21, 0.78]	0.40	**0.007**	**0.047**	
	Sexual orientation	−0.99 (0.47)	0.37	[0.15, 0.95]	0.37	**0.038**	0.152	
	Relationship	0.05 (0.32)	1.05	[0.56, 1.94]	1.05	0.884	1	
	MC-SDS	−0.46 (0.18)	0.63	[0.45, 0.89]	0.81	**0.009**	**0.047**	
								18.2^***^
(3)	CMNI-30	0.34 (0.20)	1.40	[0.94, 2.09]	1.10	0.099	0.308	
	ERQ-suppress.	0.09 (0.18)	1.09	[0.77, 1.55]	1.32	0.627	1	
	(CMNI x suppress.)	−0.36 (0.17)	0.70	[0.50, 0.97]	0.99	**0.034**	0.203	
	Age	1.09 (0.21)	2.98	[1.97, 4.50]	1.07	**<0.001**	**<0.001**	
	Education	−1.12 (0.36)	0.33	[0.16, 0.66]	0.33	**0.002**	**0.014**	
	Sexual orientation	−0.91 (0.49)	0.40	[0.15, 1.04]	0.40	0.062	0.308	
	Relationship	0.05 (0.34)	1.05	[0.54, 2.03]	1.05	0.885	1	
	MC-SDS	−0.34 (0.18)	0.71	[0.49, 1.02]	0.86	0.064	0.308	
	DV experienced	2.01 (0.35)	7.45	[3.74, 14.84]	7.45	**<0.001**	**<0.001**	
								11.8^***^

SE, standard error; OR, odds ratio; corr., adjusted for multiple testing using the Holm-method; ΔR^2^, difference of Nagelkerke’s *R*^2^ between consecutive models; MRNI-SF, Male Role Norms Inventory – Short Form; CMNI-30, Conformity to Masculine Norms Inventory – 30; ERQ-suppress., Emotion Regulation Questionnaire – Suppression subscale; MC-SDS, Marlowe-Crowne Social Desirability Scale. Reference category is non-tertiary education, heterosexual, and single. Bold formatting for significant findings on an alpha-level of 5%. ^a^Standardized coefficient; ^raw^unstandardized coefficient ^*^p < 0.05; ^**^p < 0.01; ^***^p < 0.001.

No moderation effect was found for the association between TMI and aggression in regard to alexithymia (MRNI-SF x TAS-26: *β* = 0.94, 95% CI [−0.25–2.13]; CMNI-30 x TAS-26: *β* = 1.03, 95% CI [−0.17–2.23]), cognitive reappraisal (MRNI-SF x ERQ-reappraisal: *β* = −0.24, 95% CI [−1.57–1.10]; CMNI-30 x ERQ-reappraisal: *β* = −1.11, 95% CI [−2.35–0.13]), and expressive suppression (MRNI-SF x ERQ-suppression: *β* = 0.31, 95% CI [−1.01–1.62]; CMNI-30 x ERQ-suppression: *β* < 0.01, 95% CI [−1.16–1.15]). Also, no moderation effect was found for the association between TMI and perpetration of physical DV in regard to cognitive reappraisal (MRNI-SF x ERQ-reappraisal: OR = 0.99, 95% CI [0.71–1.37]; CMNI-30 x ERQ-reappraisal: OR = 1.11, 95% CI [0.80–1.54]) and self-compassion (MRNI-SF x SCS-SF: OR = 0.84, 95% CI [0.62–1.15]; CMNI-30 x SCS-SF: OR = 1.01, 95% CI [0.73–1.40]).

## 4. Discussion

### 4.1. Summary of results

Multiple regression analyses confirmed the first two hypotheses, namely that higher levels of TMI are associated with higher levels of aggression and impaired emotional competence. In addition, conformity to but not endorsement of TMI was associated with frequent perpetration of physical DV and higher levels of alexithymia. Although significant moderation effects were found, the third hypothesis could only be partially confirmed, as the main moderation effects were contrary to our expectation. The moderation effects consisted of a buffering effect of expressive suppression on the association between both TMI measures and perpetration of DV, an analog effect of alexithymia on the association between conformity to TMI and perpetration of DV emerged, as well as a buffering effect of self-compassion on the association between endorsement of TMI and aggression was observed.

### 4.2. Integration of findings

In line with previous findings our results confirm the hypothesized association between TMI and increased aggression (Tager et al., 2010; Thomas and Levant, 2012). Following Vandello and Bosson (2013), these findings corroborate the idea that aggression is a way of demonstrating masculine status. In addition, aggression, like avoidance, may allow men to hide or mask vulnerable feelings (Berke et al., 2019). Thus, these findings further strengthen the importance of social role theories for explaining gender-based violence, namely that traditional gender ideologies may account for aggression in men, as suggested by evidence of the efficacy of gender-transformative interventions aiming to reduce violence in men (Jewkes et al., 2015).

In contrast, the hypothesized association between conformity to TMI and perpetration of DV was only significant when considering relevant sociodemographic covariates, such as age, education, and experienced physical DV. Previous evidence on a relation between TMI and IPV (Tager et al., 2010) only exists for an individual dimension of TMI (dominance) and discrepancy stress (Berke et al., 2019). Combining these findings with results from the present study, TMI seem insufficient in explaining physical DV by itself. Being an extreme form of aggression, physical DV may presuppose more than the sole endorsement of gender norms and thus be better explained by the conjunction of TMI, negative affect, and difficulties in emotion regulation (Tager et al., 2010). For example, perpetration of IPV has been related to masculine discrepancy stress (Berke et al., 2019) as an attempt to restore masculine status when men fear they fall short of masculine ideals (Bosson et al., 2009; Vandello and Bosson, 2013).

Our findings further support the proposition that conformity and endorsement of masculine ideologies are related to an impaired development of emotional competence. This was displayed by higher levels of alexithymia, more frequent use of the strategy of expressive suppression, and lower self-compassion among men with strong endorsement of and strong conformity to TMI. Notably, the effects were consistently stronger for conformity to TMI, suggesting that socially rewarding norm-conforming behavior and sanctioning non-conforming behavior (Rudman and Fairchild, 2004; Vandello and Bosson, 2013) might be particularly detrimental to men’s emotional competence. Moreover, the replication of the well-established relationship between TMI and alexithymia provides further support for the Normative Male Alexithymia Hypothesis (Levant et al., 2014) among German-speaking men. Our results therefore encourage the translation of interventions that target emotion processing and expressivity in men, such as the Alexithymia Reduction Treatment (Levant et al., 2009b).

Furthermore, in a recent study on underlying processes of alexithymia in men, difficulties in emotion processing were better explained by suppression rather than processes more closely related to trauma (Levant et al., 2014). Concordantly, in the present sample, increased endorsement of and conformity to TMI was associated with higher levels of expressive suppression, supporting this new perspective on male alexithymia and calling its traumatic etiology into question. However, contrary to our expectations, suppression did not enhance but buffer the relationship between TMI and DV. Previous research finds that expressive suppression mediates the relationship between TMI and trait aggression (Berke et al., 2020). Thus, expressive suppression may not allow to sustainably downregulate aggression but, according to our findings, may help with avoiding physical DV in a critical moment, for example, in the short time frame of a heated argument. When the suppressed affect reoccurs (i.e., a rebound effect), situational factors that may have changed and be no longer conductive of physical DV. The reoccurring aggression could then translate into alternative behaviors (e.g., kicking objects, substance abuse) or, ideally, situational changes could bring about more resources for functional affect regulation. However, because the buffering effect of expressive suppression did not remain statistically significant after controlling for covariates and multiple testing, more research on larger samples is needed to evaluate whether this is a methodological artifact, such as lacking power, or due to the absence of such an effect.

Notably, context-dependency of emotion regulation may also explain why the hypothesized relation between strong TMI and less frequent cognitive reappraisal was not confirmed. As reported in a meta-analysis by Aldao et al. (2010), many studies find negative associations between mental health related constructs, such as anxiety or depression, and functional emotion regulation (e.g., cognitive reappraisal) while finding positive associations between these constructs and dysfunctional emotion regulation (e.g., expressive suppression). Recently, a methodological reason for such findings has been proposed. In line with their results, Aldao and Nolen-Hoeksema (2012) suggest that the merit of functional emotion regulation may lie within the flexibility in reacting in line with contextual demands rather than mere frequency of use. Thus, instruments that are sensitive to contextual influences on emotion regulation efficacy may be more appropriate to investigate effects of TMI on putatively adaptive emotion regulation strategies.

When looking at functional coping strategies that are less context dependent, such as self-compassion, our results do confirm previous evidence of a negative relation between TMI and functional coping, namely strong TMI being associated with reduced self-compassion (Reilly et al., 2014). Thus, TMI may not only promote dysfunctional emotion regulation, but also undermine functional ways of coping. Our findings thus support the authors conclusion that, particularly men who would profit much from adopting self-compassion practices, may perceive mindfulness-based programs as “unmanly” (Reilly et al., 2014). Thus, it is crucial to address TMI when aiming to increase self-compassion in men, especially since the current study also indicates the possibility of a buffering effect of self-compassion on the relationship between conformity to TMI and aggression.

Lastly, another moderating effect was also found for alexithymia. Men with high levels of alexithymia showed a weaker association between strong TMI and the increased likelihood of perpetrating DV. On the one hand, this is contrasts with the hypothesis of higher emotional competence lowering negative consequences of TMI. On the other hand, a buffering effect of alexithymia on the association between TMI and DV perpetration is in line with the notion that TMI affects DV in conjunction with intense negative affect (Berke et al., 2019). Men who have higher levels of alexithymia may have difficulties recognizing, experiencing, or expressing negative affect, which, in turn, might make them less likely to act on such negative or aggressive emotions, i.e., engage in physical DV.

### 4.3. Limitations and future directions

Some limitations need to be considered when interpreting these results. First, some restraints to generalizability arise from the sample’s composition, namely psychologically distressed men, men in psychotherapy, and non-heterosexual identified men being overrepresented. Because men, especially those high in TMI, are generally more reluctant to seek help (Eggenberger et al., 2021, 2022; Walther and Seidler, 2020), the high prevalence of men using psychotherapy and being psychologically distressed in the present sample suggest a self-selection bias, as participation was based on interest in a mental health self-assessment. Similarly, the high proportion of non-heterosexual men may be explained by increased psychological burden due to the marginalization of minorities (King et al., 2008; Kelleher, 2009; Timmins et al., 2017).

Furthermore, the study’s cross-sectional design does not permit causal inference. Thus, our results cannot be interpreted in terms of causality, which may be absent or even run in the opposite direction; the same evidence could then be found if men with poor emotional competence were more willing to adhere to TMI. Nonetheless, evidence from previous studies suggests that gender-differences in emotional expressiveness only emerge gradually (Hyde, 2005). For example, Levant (1998) finds that boys lose their initial advantage in emotional expressiveness after entering preschool, where impacts through socialization are very likely. Additionally, the limited sample size – in respect to the number of simultaneous effects estimated by multiple regressions and the application of a correction for multiple testing – warrants a replication of our findings in future studies, particularly for the unexpected buffering effect of expressive suppression on the association between TMI and DV perpetration.

Some methodological restraints arise from the choice of measurements. First, psychometric properties of the German 10-item version of the MC-SDS were found to be questionable in the present sample, but comparable to other 10-item versions (e.g., Loo and Thorpe, 2000). However, this lack of reliability must be considered when drawing conclusions involving the MC-SDS. Furthermore, a recent validation study of the German version of the CMNI-30 (Komlenac et al., 2023) found that a bifactor model is only valid in heterosexual-identified men, but not sexual minority men. Hence, for about 30% of sexual minority men in the present sample, the use of a total score would not be indicated. Post-hoc sensitivity analysis showed, however, that our primary findings were comparable among the total sample and heterosexual-identified men only. Moreover, the use of unstandardized self-constructed questions to assess perpetration of DV threatens the validity of the corresponding results, and their broad formulation reduces sensitivity to differences in DV. Despite the definition of DV including both violence towards a partner or child, different risk factors have been identified (Klevens and Whitaker, 2007; Capaldi et al., 2012). By grouping violence against partners and children together, our measures cannot account for potential differences in the association with TMI and emotional competence. However, violence against partners and children often co-occur, and have been identified as risk-factors for one another (Smith Slep and Heyman, 2001). A strength of the broad formulation of the questions assessing DV is that it may have lowered the hurdle for admitting DV. Furthermore, all calculations were controlled for social desirability and experienced victimization by partners and children. Another methodological limitation is that the TMI measures used in the present study did not account for discrepancy stress, which has previously been suggested to better explain DV (Reidy et al., 2014; Berke et al., 2019). Furthermore, the instruments assessing emotion regulation focused solely on the frequency of application while not accounting for the flexibility in accurately reacting to contextual demands. Future studies should also include instruments accounting for the context-dependency of emotion regulation efficacy.

Finally, on a broader theoretical level, it can be criticized that the study focuses solely on social gender role norms while neglecting biological and intersectional perspectives. However, our argumentation does not exclude biological factors, and may contribute to intersectional explanations of violence. For example, when considering poverty, an established risk-factor of DV. Because difficulties in providing for one’s family may be perceived as a threat to masculine status, gender ideology can be seen as a crucial variable in this context (Bosson et al., 2009; Vandello and Bosson, 2013). Future studies that use an intersectional perspective on DV may therefore consider including potential effects of gender role ideologies into their design.

### 4.4. Prevention and clinical implications

The efficacy of gender-transformative interventions and training male DV perpetrator in various emotional competence dimensions, such as increasing empathy in order to compensate for potential developmental deficits in emotional competence, to prevent physical DV in men are well documented (Pickover, 2010; Jewkes et al., 2015). As our study replicated the associations between strong TMI, increased aggression, and impaired emotional control, as well as conformity to TMI and more frequent perpetration of physical DV among a sample of German-speaking men, we highly encourage the translation of interventions aimed at emotional competence, such as the Alexithymia Reduction Treatment (Levant et al., 2009b), into the German language. Furthermore, following the findings and theories about the role of emotional competence in male psychopathology (e.g., Aldao et al., 2010), our findings not only support the implementation of gender-transformative interventions to reduce aggression and physical DV, but also in general psychotherapy.

## 5. Conclusion

The present study highlights the relevance of socially constructed gender ideologies, such as TMI, as important constructs related to aggression, perpetration of DV, and impaired emotional competence in men from German-speaking parts of Europe. On the one hand, these results empirically underscore the necessity to address and deconstruct TMI in interventions targeting aggression, perpetration of physical DV, and emotional competence in men, ideally replacing TMI with functional alternative masculinities. On the other hand, they encourage the transfer of interventions designed for men with strong TMI into the German language such as the Alexithymia Reduction Treatment (Levant et al., 2009b). Furthermore, in the context of DV perpetration, expressive suppression might serve as a functional emotion regulation strategy for men, particularly those with strong TMI, in emotionally charged situations. However, future studies using experimental designs, including measures of discrepancy stress, and considering the context-dependent nature of emotional competence are needed to gain deeper insight into the role of TMI in gender-based aggression and physical DV.

## Data availability statement

The raw data supporting the conclusions of this article will be made available by the authors, without undue reservation.

## Ethics statement

The studies involving human participants were reviewed and approved by Ethics Committee of the Faculty of Philosophy of the University of Zurich. The patients/participants provided their written informed consent to participate in this study.

## Author contributions

AW: conceptualization and Supervision. AW, FL, and LE: methodology, formal analysis, investigation, and writing–original draft preparation. AW, FL, LE, NK, MS, and UE: writing–review and editing. AW and UE: funding acquisition and resources. All authors contributed to the article and approved the submitted version.

## Funding

This research was funded by the Swiss National Science Foundation (Grant PZPGP1_201757 awarded to AW). AW and LE are supported by a grant from the Swiss National Science Foundation (Grant PZPGP1_201757 awarded to AW). Open Access costs financed by University Library Zurich.

## Conflict of interest

The authors declare that the research was conducted in the absence of any commercial or financial relationships that could be construed as a potential conflict of interest.

## Publisher’s note

All claims expressed in this article are solely those of the authors and do not necessarily represent those of their affiliated organizations, or those of the publisher, the editors and the reviewers. Any product that may be evaluated in this article, or claim that may be made by its manufacturer, is not guaranteed or endorsed by the publisher.
