# Supplementary material for: How do traditional masculinity ideologies and emotional competence relate to aggression and physical domestic violence in cisgender men?
Source: Front Psychol. 2023 Mar 14;14:1100114. doi: 10.3389/fpsyg.2023.1100114 (PMC10043379; doi:10.3389/fpsyg.2023.1100114)
Supplement: Supplementary file 1 [file Data_Sheet_1.docx]

**How do Traditional Masculinity Ideologies and Emotional Competence relate to Aggression and Physical Domestic Violence in Cisgender Men?**

**Supplementary Materials**

**Content**

| **Table S1.** | *Overview of the Sociodemographic Questions (Q), Answer Options (A), and Groupings (Grp.)* | page 2 |
| --- | --- | --- |
| **Table S2.** | *Psychometric Properties of the Questionnaires* | page 3 |
| **Figure S1.** | *Empirical and Theoretical Normal Density of the Questionnaires* | page 4 |
| **Table S3.** | *Pearson's Correlation Coefficients Including Subscales* | page 5 |
| **Table S4.** | *Sensitivity Analyses for Linear Regression Models with Aggression (ABPQ) as Outcome Variable* | page 6 |
| **Table S5.** | *Sensitivity Analyses for Binomial Logistic Regression Models with DV Perpetration as Outcome Variable* | page 7 |
| **Table S6.** | *Linear Regression Models with Emotion Suppression (ERQ-suppression) as Outcome Variable* | page 8 |
| **Table S7.** | *Linear Regression Models with Self-Compassion (SCS-SF) as Outcome Variable* | page 9 |
| **Table S8.** | *Linear Regression Models with Alexithymia (TAS-26) as Outcome Variable* | page 10 |
| **Table S9.** | *Linear Regression Models with Cognitive Reappraisal (ERQ-reappraisal) as Outcome Variable* | page 11 |
| **Table S10.** | *Moderation Models with Aggression (ABPQ) as Outcome and Self-Compassion (SCS-SF) as Moderator* | page 12 |
| **Table S11.** | *Moderation Models with DV Perpetration as Outcome and Alexithymia (TAS-26) as Moderator* | page 14 |
| **Table S12.** | *Sensitivity Analyses for Moderation Models with DV Perpetration as Outcome and Expressive Suppression as Moderator* | page 16 |

| **Table S1** *Overview of the Sociodemographic Questions (Q), Answer Options (A), and Groupings (Grp.)* | |
| --- | --- |
| Q*:* | “What is your current gender identity?” |
| A: | 1 = *male*; 2 = *female*; 3 = *female-to-male transgender*; 4 = *male-to-female-transgender;* 5 = *gender neutral / non-binary;* 6 = *diverse*; 7 = *other* (with a free-text option); 8 = *don’t know / not sure* |
| Q: | “What sex were you assigned at birth ("biological sex")?” |
| A: | 1 = *male*; 2 = *female*; 3 = *diverse*; 4 = *other* (with a free-text option); 5 = *don’t know / not sure* |
| Q: | “Please enter your age in years (e.g., 42).” |
| A: | free-text option |
| Q: | “What is your highest completed education?” |
| A: | 1 = *no completed education*; 2 = *special school*; 3 = *secondary school*;  4 = *high school* ("Realschule"); 5 = *basic apprenticeship*; 6 = *apprenticeship*;  7 = *gymnasium* ("Abitur"); 8 = *university*; 9 = *other* |
| Grp.: | 1 = *none completed*; 2 = *secondary education*; 3 = *tertiary education*; 4 = *other* |
| Q: | “Which nationality do you belong to?” |
| A: | 1 = *Swiss*; 2 = *German*; 3 = *Austrian*; 4 = *Liechtensteiner*; 5 = *Luxembourger*;  6 = *Belgian*; 7 = *other* |
| Q: | “What is your sexual orientation?” |
| A: | 1 = *heterosexual*; 2 = *gay / lesbian*; 3 = *bisexual*; 4 = *asexual*; 5 = *other* (with a free-text option); 6 = *don’t know / not sure* |
| Grp.: | 1 = *heterosexual*; 2 = *non-heterosexual* |
| Q: | “What is your marital status?” |
| A: | 1 = *unmarried (single)*; 2 = *unmarried (in a relationship)*; 3 = *married*;  4 = *in a registered partnership*; 5 = *divorced*; 6 = *widowed* |
| Grp.: | 1 = *unmarried*; 2 = *married / registered partnership*; 3 = *separated* |
| Q: | “Are you currently in an intimate relationship?” |
| A: | 1 = *yes*; 2 = *no*; 3 = *yes, but not in an exclusive relationship* |
| Q: | "Are you currently suffering from a diagnosed acute or chronic mental disorder?" |
| A: | 1 = *yes*; 2 = *no* |
| Q: | "Are you currently experiencing psychological distress?" |
| A: | 1 = *yes*; 2 = *no* |
| Q: | "Are you currently receiving psychotherapeutic treatment?" |
| A: | 1 = *yes*; 2 = *no* |
| *Note*. *Q* = Question; *A* = Answer options; *Grp.* = Groupings used for the analyses | |

| **Table S2** *Psychometric Properties of the Questionnaires* | | | | | | | |
| --- | --- | --- | --- | --- | --- | --- | --- |
| Questionnaire | items | α | ω | mean (SD) | range | skewness | kurtosis |
| MRNI-SF | 21 | .94 | .95 | 46.7 (21.0) | [21; 138] | 1.01 | 0.90 |
| CMNI-30 | 30 | .85 | .88 | 53.2 (17.7) | [11; 134] | 0.73 | 1.37 |
| TAS-26 | 18 | .83 | .88 | 45.8 (10.5) | [18; 80] | 0.07 | -0.38 |
| ERQ-reappraisal | 6 | .73 | .83 | 25.1 (6.5) | [6; 42] | -0.29 | 0.01 |
| ERQ-suppression | 4 | .63 | .70 | 15.5 (4.2) | [4; 28] | -0.06 | -0.20 |
| SCS-SF | 12 | .89 | .91 | 36.6 (9.4) | [14; 59] | -0.04 | -0.65 |
| BPAQ | 29 | .91 | .92 | 39.2 (17.9) | [6; 95] | 0.55 | -0.22 |
| MC-SDS | 10 | .61 | .67 | 4.3 (2.2) | [0; 10] | 0.10 | -0.52 |
| Note. items = number of items; α = Cronbach’s Alpha; ω = McDonald’s Omega | | | | | | | |

| **Figure S1** *Empirical and Theoretical Normal Density of the Questionnaires* |
| --- |
| 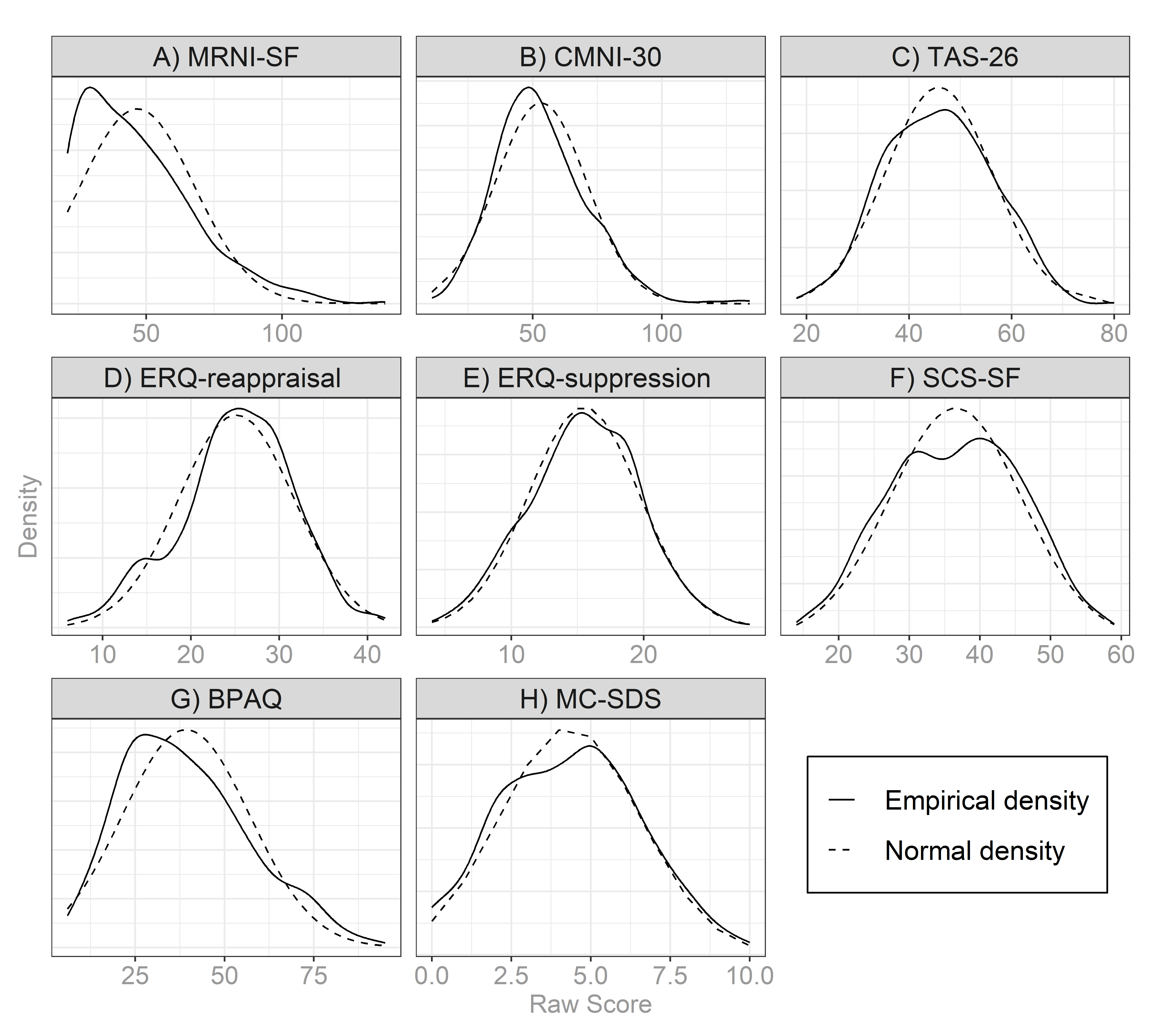 |
| *Note.* MRNI-SF = Male Role Norms Inventory – Short Form; CMNI-30 = Conformity to Masculine Norms Inventory – 30; TAS-26 = Toronto Alexithymia Scale – 26; ERQ = Emotion Regulation Questionnaire; SCS-SF = Self-Compassion Scale – Short Form; BPAQ = Buss-Perry Aggression Questionnaire; MC-SDS = Marlowe-Crowne Social Desirability Scale |

| **Table S3** *Pearson's Correlation Coefficients Inclduing Subscales* | | | | | | | | | | | | | | | | | | |
| --- | --- | --- | --- | --- | --- | --- | --- | --- | --- | --- | --- | --- | --- | --- | --- | --- | --- | --- |
|  | 1 | 2 | 2.1 | 2.2 | 2.3 | 2.4 | 2.5 | 2.6 | 2.7 | 2.8 | 2.9 | 2.10 | 3 | 4.1 | 4.2 | 5 | 6 | 7 |
| 1. MRNI-SF | – |  |  |  |  |  |  |  |  |  |  |  |  |  |  |  |  |  |
| 2. CMNI-30 | **.58^***^** | – |  |  |  |  |  |  |  |  |  |  |  |  |  |  |  |  |
| 2.1 Emotional Control | **.24^***^** | **.46^***^** | – |  |  |  |  |  |  |  |  |  |  |  |  |  |  |  |
| 2.2 Winning | **.31^***^** | **.65^***^** | .15 | – |  |  |  |  |  |  |  |  |  |  |  |  |  |  |
| 2.3 Playboy | **.18^*^** | **.46^***^** | .04 | **.18^*^** | – |  |  |  |  |  |  |  |  |  |  |  |  |  |
| 2.4 Violence | **.35^***^** | **.55^***^** | .15 | **.31^***^** | **.18^*^** | – |  |  |  |  |  |  |  |  |  |  |  |  |
| 2.5 Heterosexuality | **.51^***^** | **.54^***^** | .16 | **.29^***^** | .07 | **.19^*^** | – |  |  |  |  |  |  |  |  |  |  |  |
| 2.6 Status | .13 | **.50^***^** | .04 | **.38^***^** | **.20^**^** | **.25^***^** | .15 | – |  |  |  |  |  |  |  |  |  |  |
| 2.7 Work | **.18^*^** | **.46^***^** | .16 | **.24^***^** | .10 | .14 | .05 | **.23^***^** | – |  |  |  |  |  |  |  |  |  |
| 2.8 Power | **.65^***^** | **.58^***^** | **.18^*^** | **.34^***^** | **.22^***^** | **.27^***^** | **.37^***^** | **.22^***^** | .15 | – |  |  |  |  |  |  |  |  |
| 2.9 Self-Reliance | **.20^**^** | **.49^***^** | **.37^***^** | **.22^***^** | .09 | .13 | **.21^**^** | .04 | .13 | **.17^*^** | – |  |  |  |  |  |  |  |
| 2.10 Rsik-Taking | **.23^***^** | **.42^***^** | -.01 | **.35^***^** | **.17^*^** | **.22^***^** | .12 | .14 | **.19^**^** | .08 | .12 | – |  |  |  |  |  |  |
| 3. TAS-26 | .06 | **.25^***^** | **.50^***^** | .05 | .00 | .05 | .11 | -.10 | .11 | .08 | **.43^***^** | -.02 | – |  |  |  |  |  |
| 4.1 ERQ-reappraisal | .02 | -.01 | -.05 | .08 | -.05 | -.06 | .05 | -.02 | -.02 | .07 | -.13 | .12 | **-.25^***^** | – |  |  |  |  |
| 4.2 ERQ-suppression | **.20^**^** | **.34^***^** | **.55^***^** | .16 | -.02 | .10 | **.20^**^** | -.07 | **.19^**^** | .16 | **.33^***^** | .06 | **.44^***^** | **.31^***^** | – |  |  |  |
| 5. SCS-SF | -.16 | **-.36^***^** | **-.31^***^** | **-.18^*^** | -.11 | -.16 | **-.20^**^** | -.10 | -.14 | **-.17^*^** | **-.44^***^** | -.01 | **-.60^***^** | **.41^***^** | **-.20^**^** | – |  |  |
| 6. BPAQ | **.35^***^** | **.48^***^** | .11 | **.39^***^** | **.20^**^** | **.45^***^** | **.24^***^** | .11 | **.19^**^** | **.25^***^** | **.32^***^** | **.28^***^** | **.31^***^** | **-.22^***^** | .15 | **-.51^***^** | – |  |
| 7. MC-SDS | -.14 | **-.37^***^** | -.06 | **-.36^***^** | **-.23^***^** | **-.28^***^** | -.14 | **-.27^***^** | -.10 | **-.19^**^** | -.14 | **-.18^*^** | **-.17^*^** | .11 | -.04 | **.34^***^** | **-.51^***^** | – |
| 8. DV perpetration | .10 | .08 | -.02 | .06 | .02 | .11 | .02 | -.02 | .08 | .08 | .04 | .04 | .04 | -.04 | .01 | -.03 | **.21^**^** | -.11 |
| Note. *p*-values were adjusted for multiple testing using the Holm-method; MRNI-SF = Male Role Norms Inventory – Short Form; CMNI-30 = Conformity to Masculine Norms Inventory – 30; TAS-26 = Toronto Alexithymia Scale – 26; ERQ = Emotion Regulation Questionnaire; SCS-SF = Self-Compassion Scale – Short Form; BPAQ = Buss-Perry Aggression Questionnaire; MC-SDS = Marlowe-Crowne Social Desirability Scale; DV = domestic violence (0 = no perpetration, 1 = perpetration). Bold formatting for statistical significance on an alpha-level of 5%. ^*^ *p* < .05; ^**^ *p* < .01; ^***^ p < .001 | | | | | | | | | | | | | | | | | | |

| **Table S4** *Sensitivity Analyses for Linear Regression Models with Aggression (ABPQ) as Outcome Variable* | | | | | | | |
| --- | --- | --- | --- | --- | --- | --- | --- |
| Predictor | | β^a^ (SE) | 95% CI | B^raw^ | *p* | *p* (corr.) | Δ*R*^2^ (%) |
| A) Total Sample | | | | | | | |
| 1) | CMNI-30 | 8.66 (0.76) | [7.17, 10.14] | 0.49 | **<.001** | – |  |
|  |  |  |  |  |  |  | **23.3^***^** |
| 2) | CMNI-30 | 6.44 (0.74) | [4.99, 7.89] | 0.36 | **<.001** | **<.001** |  |
|  | Age | 1.80 (0.73) | [0.36, 3.24] | 0.12 | **.015** | **.029** |  |
|  | Education | -4.80 (1.44) | [-7.63, -1.97] | -4.80 | **<.001** | **.004** |  |
|  | Sexual orientation | 2.02 (1.61) | [-1.16, 5.19] | 2.02 | .212 | .212 |  |
|  | Relationship | -4.53 (1.38) | [-7.25, -1.82] | -4.53 | **.001** | **.004** |  |
|  | MC-SDS | -6.92 (0.73) | [-8.35, -5.49] | -3.12 | **<.001** | **<.001** |  |
|  |  |  |  |  |  |  | **16.1^***^** |
| 3) | CMNI-30 | 6.34 (0.73) | [4.89, 7.78] | 0.36 | **<.001** | **<.001** |  |
|  | Age | 1.63 (0.73) | [0.19, 3.06] | 0.11 | **.026** | .053 |  |
|  | Education | -4.84 (1.43) | [-7.65, -2.04] | -4.84 | **<.001** | **.004** |  |
|  | Sexual orientation | 2.22 (1.60) | [-0.93, 5.38] | 2.22 | .166 | .166 |  |
|  | Relationship | -4.55 (1.37) | [-7.24, -1.86] | -4.55 | **<.001** | **.004** |  |
|  | MC-SDS | -6.66 (0.73) | [-8.09, -5.23] | -3.00 | **<.001** | **<.001** |  |
|  | DV experienced | 5.18 (1.83) | [1.58, 8.79] | 5.18 | **.005** | **.015** |  |
|  |  |  |  |  |  |  | **1.0^**^** |
| B) Heterosexual-Identified Men | | | | | | | |
| 1) | CMNI-30 | 7.97 (0.87) | [6.25 , 9.69] | 0.46 | **< .001** | – |  |
|  |  |  |  |  |  |  | **20.1^***^** |
| 2) | CMNI-30 | 5.9 (0.86) | [4.21 , 7.59] | 0.34 | **<.001** | **<.001** |  |
|  | Age | 2.52 (0.85) | [0.85 , 4.19] | 0.16 | **.003** | **.006** |  |
|  | Education | -5.64 (1.68) | [-8.94 ,-2.34] | -5.64 | **<.001** | **.003** |  |
|  | Relationship | -3.45 (1.6) | [-6.61 ,-0.30] | -3.45 | **.032** | **.032** |  |
|  | MC-SDS | -7.01 (0.84) | [-8.67 ,-5.36] | -3.21 | **<.001** | **<.001** |  |
|  |  |  |  |  |  |  | **16.6^***^** |
| 3) | CMNI-30 | 5.79 (0.86) | [4.11 , 7.48] | 0.34 | **<.001** | **<.001** |  |
|  | Age | 2.35 (0.85) | [0.68 , 4.02] | 0.15 | **.006** | **.018** |  |
|  | Education | -5.74 (1.67) | [-9.02 ,-2.45] | -5.74 | **<.001** | **.003** |  |
|  | Relationship | -3.4 (1.59) | [-6.54 ,-0.27] | -3.40 | **.034** | .063 |  |
|  | MC-SDS | -6.76 (0.85) | [-8.42 ,-5.10] | -3.10 | **<.001** | **<.001** |  |
|  | DV experienced | 4.46 (2.07) | [0.39 , 8.54] | 4.46 | **.032** | .063 |  |
|  |  |  |  |  |  |  | **0.7^*^** |
| *Note.* SE = standard error; corr. = adjusted for multiple testing using the Holm-method; Δ*R*^2^ = difference of adjusted *R*^2^ between consecutive models; CMNI-30 = Conformity to Masculine Norms Inventory – 30; MC-SDS = Marlowe-Crowne Social Desirability Scale. Reference category is non-tertiary education, heterosexual, and single. Bold formatting for significant findings on an alpha-level of 5%. ^a^ standardized coefficient; ^raw^ unstandardized coefficient ^*^ *p* < .05; ^**^ *p* < .01; ^***^ p < .001 | | | | | | | |

| **Table S5** *Sensitivity Analyses for Binomial Logistic Regression Models with DV Perpetration as Outcome Variable* | | | | | | | | |
| --- | --- | --- | --- | --- | --- | --- | --- | --- |
| Predictor | | β^a^ (SE) | OR^a^ | 95% CI | OR^raw^ | *p* | *p* (corr.) | Δ*R*^2^ (%) |
| A) Total Sample | | | | | | | | |
| 1) | CMNI-30 | 0.22 (0.13) | 1.25 | [0.96, 1.63] | 1.01 | .093 | – |  |
|  |  |  |  |  |  |  |  | 1.2 |
| 2) | CMNI-30 | 0.34 (0.16) | 1.40 | [1.01, 1.93] | 1.02 | **.042** | .103 |  |
|  | Age | 1.03 (0.19) | 2.81 | [1.92, 4.10] | 1.07 | **<.001** | **<.001** |  |
|  | Education | -1.01 (0.33) | 0.36 | [0.19, 0.69] | 0.36 | **.002** | **.011** |  |
|  | Sexual orientation | -0.99 (0.47) | 0.37 | [0.15, 0.93] | 0.37 | **.034** | .103 |  |
|  | Relationship | 0.04 (0.31) | 1.04 | [0.56, 1.91] | 1.04 | .903 | .903 |  |
|  | MC-SDS | -0.41 (0.17) | 0.66 | [0.47, 0.92] | 0.83 | **.014** | .056 |  |
|  |  |  |  |  |  |  |  | **18.8^***^** |
| 3) | CMNI-30 | 0.37 (0.18) | 1.44 | [1.00, 2.08] | 1.02 | **.048** | .190 |  |
|  | Age | 1.09 (0.21) | 2.96 | [1.96, 4.47] | 1.07 | **<.001** | **<.001** |  |
|  | Education | -1.20 (0.36) | 0.30 | [0.15, 0.61] | 0.30 | **<.001** | **.004** |  |
|  | Sexual orientation | -0.91 (0.48) | 0.40 | [0.16, 1.04] | 0.40 | .060 | .190 |  |
|  | Relationship | 0.04 (0.33) | 1.04 | [0.54, 2.00] | 1.04 | .907 | .907 |  |
|  | MC-SDS | -0.29 (0.18) | 0.75 | [0.53, 1.06] | 0.88 | .106 | .212 |  |
|  | DV experienced | 2.08 (0.35) | 7.98 | [4.04, 15.78] | 7.98 | **<.001** | **<.001** |  |
|  |  |  |  |  |  |  |  | **13.2^***^** |
| B) Heterosexual-Identified Men | | | | | | | | |
| 1) | CMNI-30 | 0.24 (0.15) | 1.27 | [0.95, 1.69] | 1.01 | .102 | .102 |  |
|  |  |  |  |  |  |  |  | 0.1 |
| 2) | CMNI-30 | 0.41 (0.18) | 1.51 | [1.07, 2.14] | 1.02 | **.019** | .058 |  |
|  | Age | 1.06 (0.21) | 2.89 | [1.91, 4.36] | 1.07 | **<.001** | **<.001** |  |
|  | Education | -1.18 (0.36) | 0.31 | [0.15, 0.62] | 0.31 | **.001** | **.004** |  |
|  | Relationship | 0.21 (0.34) | 1.24 | [0.64, 2.40] | 1.24 | .526 | .526 |  |
|  | MC-SDS | -0.39 (0.18) | 0.68 | [0.48, 0.96] | 0.84 | **.029** | .058 |  |
|  |  |  |  |  |  |  |  | **18.3^***^** |
| 3) | CMNI-30 | 0.42 (0.19) | 1.53 | [1.04, 2.23] | 1.02 | **.029** | .088 |  |
|  | Age | 1.07 (0.22) | 2.93 | [1.89, 4.53] | 1.07 | **<.001** | **<.001** |  |
|  | Education | -1.37 (0.39) | 0.25 | [0.12, 0.54] | 0.25 | **<.001** | **.002** |  |
|  | Relationship | 0.25 (0.36) | 1.28 | [0.63, 2.58] | 1.28 | .493 | .493 |  |
|  | MC-SDS | -0.26 (0.19) | 0.77 | [0.53, 1.12] | 0.89 | .169 | .337 |  |
|  | DV experienced | 1.92 (0.37) | 6.82 | [3.28, 14.15] | 6.82 | **<.001** | **<.001** |  |
|  |  |  |  |  |  |  |  | **11.9^***^** |
| *Note.* SE = standard error; OR = odds ratio; corr. = adjusted for multiple testing using the Holm-method; Δ*R*^2^ = difference of Nagelkerke’s R^2^ between consecutive models; MRNI-SF = Male Role Norms Inventory – Short Form; CMNI-30 = Conformity to Masculine Norms Inventory – 30; MC-SDS = Marlowe-Crowne Social Desirability Scale. Reference category is non-tertiary education, heterosexual, and single. Bold formatting for significant findings on an alpha-level of 5%. ^a^ standardized coefficient; ^raw^ unstandardized coefficient ^*^ *p* < .05; ^**^ *p* < .01; ^***^ p < .001 | | | | | | | | |

| **Table S6** *Linear Regression Models with Emotion Suppression (ERQ-suppression) as Outcome Variable* | | | | | | | |
| --- | --- | --- | --- | --- | --- | --- | --- |
|  | Predictor | β^a^ (SE) | 95% CI | B^raw^ | *p* | *p* (corr.) | Δ*R*^2^ (%) |
| A) Endorsement of TMI as Predictor | | | | | | | |
| 1) | MRNI-SF | 0.82 (0.20) | [0.43, 1.21] | 0.04 | **<.001** | – |  |
|  |  |  |  |  |  |  | **2.2^***^** |
| 2) | MRNI-SF | 0.92 (0.20) | [0.52, 1.31] | 0.04 | **<.001** | **<.001** |  |
|  | Age | -0.63 (0.21) | [-1.05, -0.22] | -0.04 | **.003** | **.014** |  |
|  | Education | -0.40 (0.42) | [-1.22, 0.42] | -0.40 | .343 | 1 |  |
|  | Sexual orientation | 0.38 (0.47) | [-0.55, 1.31] | 0.38 | .425 | 1 |  |
|  | Relationship | -0.40 (0.40) | [-1.19, 0.38] | -0.40 | .313 | 1 |  |
|  | MC-SDS | 0.03 (0.20) | [-0.36, 0.42] | 0.01 | .869 | 1 |  |
|  |  |  |  |  |  |  | **1.5^**^** |
| B) Conformity to TMI as Predictor | | | | | | | |
| 1) | CMNI-30 | 1.40 (0.19) | [1.03, 1.78] | 0.08 | **< .001** | – |  |
|  |  |  |  |  |  |  | **5.7^***^** |
| 2) | CMNI-30 | 1.51 (0.21) | [1.10, 1.92] | 0.08 | **<.001** | **<.001** |  |
|  | Age | -0.29 (0.21) | [-0.69, 0.12] | -0.02 | .163 | .654 |  |
|  | Education | -0.51 (0.40) | [-1.30, 0.28] | -0.51 | .206 | .654 |  |
|  | Sexual orientation | 0.32 (0.45) | [-0.56, 1.22] | 0.32 | .473 | .654 |  |
|  | Relationship | -0.45 (0.39) | [-1.21, 0.31] | -0.45 | .241 | .654 |  |
|  | MC-SDS | 0.42 (0.20) | [0.02, 0.82] | 0.19 | **.038** | .189 |  |
|  |  |  |  |  |  |  | **0.9^*^** |
| *Note.* SE = standard error; corr. = adjusted for multiple testing using the Holm-method; Δ*R*^2^ = difference of adjusted *R*^2^ between consecutive models; MRNI-SF = Male Role Norms Inventory – Short Form; CMNI-30 = Conformity to Masculine Norms Inventory – 30; MC-SDS = Marlowe-Crowne Social Desirability Scale. Reference category is non-tertiary education, heterosexual, and single. Bold formatting for significant findings on an alpha-level of 5%. ^a^ standardized coefficient; ^raw^ unstandardized coefficient ^*^ *p* < .05; ^**^ *p* < .01; ^***^ p < .001 | | | | | | | |

| **Table S7** *Linear Regression Models with Self-Compassion (SCS-SF) as Outcome Variable* | | | | | | | |
| --- | --- | --- | --- | --- | --- | --- | --- |
|  | Predictor | β^a^ (SE) | 95% CI | B^raw^ | *p* | *p* (corr.) | Δ*R*^2^ (%) |
| A) Endorsement of TMI as Predictor | | | | | | | |
| 1) | MRNI-SF | -1.48 (0.45) | [-2.36, -0.59] | -0.07 | **.001** | – |  |
|  |  |  |  |  |  |  | **1.8^**^** |
| 2) | MRNI-SF | -1.17 (0.42) | [-2.00, -0.34] | -0.06 | **.006** | **.024** |  |
|  | Age | 1.17 (0.44) | [0.30, 2.04] | 0.08 | **.008** | **.025** |  |
|  | Education | 0.10 (0.88) | [-1.62, 1.83] | 0.10 | .906 | 1 |  |
|  | Sexual orientation | -0.44 (0.99) | [-2.40, 1.51] | -0.44 | .659 | 1 |  |
|  | Relationship | 3.81 (0.84) | [2.15, 5.47] | 3.81 | **<.001** | **<.001** |  |
|  | MC-SDS | 2.73 (0.42) | [1.90, 3.55] | 1.23 | **<.001** | **<.001** |  |
|  |  |  |  |  |  |  | **12.7^***^** |
| B) Conformity to TMI as Predictor | | | | | | | |
| 1) | CMNI-30 | -3.41 (0.42) | [-4.24, -2.57] | -0.19 | **<.001** | – |  |
|  |  |  |  |  |  |  | **10.4^***^** |
| 2) | CMNI-30 | -2.43 (0.44) | [-3.29, -1.56] | -0.14 | **<.001** | **<.001** |  |
|  | Age | 0.64 (0.44) | [-0.22, 1.50] | 0.04 | .144 | .431 |  |
|  | Education | 0.33 (0.86) | [-1.36, 2.01] | 0.33 | .704 | 1 |  |
|  | Sexual orientation | -0.47 (0.96) | [-2.36, 1.42] | -0.47 | .625 | 1 |  |
|  | Relationship | 3.86 (0.82) | [2.24, 5.47] | 3.86 | **<.001** | **<.001** |  |
|  | MC-SDS | 2.06 (0.43) | [1.21, 2.91] | 0.93 | **<.001** | **<.001** |  |
|  |  |  |  |  |  |  | **7.4^***^** |
| *Note.* SE = standard error; corr. = adjusted for multiple testing using the Holm-method; Δ*R*^2^ = difference of adjusted *R*^2^ between consecutive models; MRNI-SF = Male Role Norms Inventory – Short Form; CMNI-30 = Conformity to Masculine Norms Inventory – 30; MC-SDS = Marlowe-Crowne Social Desirability Scale. Reference category is non-tertiary education, heterosexual, and single. Bold formatting for significant findings on an alpha-level of 5%. ^a^ standardized coefficient; ^raw^ unstandardized coefficient ^*^ *p* < .05; ^**^ *p* < .01; ^***^ p < .001 | | | | | | | |

| **Table S8** *Linear Regression Models with Alexithymia (TAS-26) as Outcome Variable* | | | | | | | |
| --- | --- | --- | --- | --- | --- | --- | --- |
|  | Predictor | β^a^ (SE) | 95% CI | B^raw^ | *p* | *p* (corr.) | Δ*R*^2^ (%) |
| A) Endorsement of TMI as Predictor | | | | | | | |
| 1) | MRNI-SF | 0.67 (0.51) | [-0.33, 1.66] | 0.03 | .189 | .189 |  |
|  |  |  |  |  |  |  | < 0.1 |
| 2) | MRNI-SF | 0.53 (0.50) | [-0.46, 1.51] | 0.02 | .292 | .585 |  |
|  | Age | -0.99 (0.52) | [-2.01, 0.04] | -0.06 | .059 | .177 |  |
|  | Education | -2.20 (1.04) | [-4.24, -0.17] | -2.20 | **.034** | .136 |  |
|  | Sexual orientation | -0.73 (1.17) | [-3.03, 1.58] | -0.73 | .536 | .585 |  |
|  | Relationship | -3.75 (0.99) | [-5.71, -1.80] | -3.75 | **<.001** | **.001** |  |
|  | MC-SDS | -1.45 (0.49) | [-2.42, -0.47] | -0.65 | **.004** | **.018** |  |
|  |  |  |  |  |  |  | **1.7^***^** |
| B) Conformity to TMI as Predictor | | | | | | | |
| 1) | CMNI-30 | 2.61 (0.49) | [1.64, 3.57] | 0.15 | **< .001** | **< .001** |  |
|  |  |  |  |  |  |  | **1.2^***^** |
| 2) | CMNI-30 | 2.10 (0.52) | [1.07, 3.13] | 0.12 | **<.001** | **<.001** |  |
|  | Age | -0.57 (0.52) | [-1.59, 0.45] | -0.04 | .273 | .546 |  |
|  | Education | -2.46 (1.02) | [-4.46, -0.45] | -2.46 | **.016** | .066 |  |
|  | Sexual orientation | -0.52 (1.14) | [-2.76, 1.73] | -0.52 | .651 | .651 |  |
|  | Relationship | -3.74 (0.98) | [-5.66, -1.82] | -3.74 | **<.001** | **<.001** |  |
|  | MC-SDS | -0.80 (0.51) | [-1.81, 0.21] | -0.36 | .121 | .364 |  |
|  |  |  |  |  |  |  | **1.2^***^** |
| *Note.* SE = standard error; corr. = adjusted for multiple testing using the Holm-method; Δ*R*^2^ = difference of adjusted *R*^2^ between consecutive models; MRNI-SF = Male Role Norms Inventory – Short Form; CMNI-30 = Conformity to Masculine Norms Inventory – 30; MC-SDS = Marlowe-Crowne Social Desirability Scale. Reference category is non-tertiary education, heterosexual, and single. Bold formatting for significant findings on an alpha-level of 5%. ^a^ standardized coefficient; ^raw^ unstandardized coefficient ^*^ *p* < .05; ^**^ *p* < .01; ^***^ p < .001 | | | | | | | |

| **Table S9** *Linear Regression Models with Cognitive Reappraisal (ERQ-reappraisal) as Outcome Variable* | | | | | | | |
| --- | --- | --- | --- | --- | --- | --- | --- |
|  | Predictor | β^a^ (SE) | 95% CI | B^raw^ | *p* | *p* (corr.) | Δ*R*^2^ (%) |
| A) Endorsement of TMI as Predictor | | | | | | | |
| 1) | MRNI-SF | 0.14 (0.32) | [-0.48, 0.76] | 0.01 | .654 | .654 |  |
|  |  |  |  |  |  |  | < 0.1 |
| 2) | MRNI-SF | 0.33 (0.32) | [-0.30, 0.97] | 0.02 | .304 | .875 |  |
|  | Age | -1.06 (0.34) | [-1.73, -0.40] | -0.07 | **.002** | **.010** |  |
|  | Education | 0.81 (0.67) | [-0.51, 2.12] | 0.81 | .227 | .875 |  |
|  | Sexual orientation | 0.02 (0.76) | [-1.46, 1.51] | 0.02 | .974 | .974 |  |
|  | Relationship | 0.79 (0.64) | [-0.47, 2.05] | 0.79 | .219 | .875 |  |
|  | MC-SDS | 0.85 (0.32) | [0.23, 1.48] | 0.38 | **.008** | **.039** |  |
|  |  |  |  |  |  |  | **1.0^**^** |
| B) Conformity to TMI as Predictor | | | | | | | |
| 1) | CMNI-30 | -0.07 (0.32) | [-0.70, 0.55] | -0.00 | .815 | .815 |  |
|  |  |  |  |  |  |  | < 0.1 |
| 2) | CMNI-30 | 0.04 (0.34) | [-0.64, 0.71] | 0.00 | .912 | 1 |  |
|  | Age | -1.03 (0.34) | [-1.70, -0.36] | -0.07 | **.003** | **.016** |  |
|  | Education | 0.84 (0.67) | [-0.47, 2.16] | 0.84 | .209 | .835 |  |
|  | Sexual orientation | -0.09 (0.75) | [-1.57, 1.38] | -0.09 | .902 | 1 |  |
|  | Relationship | 0.76 (0.64) | [-0.51, 2.02] | 0.76 | .240 | .835 |  |
|  | MC-SDS | 0.82 (0.34) | [0.16, 1.49] | 0.37 | **.015** | .077 |  |
|  |  |  |  |  |  |  | **0.9^**^** |
| *Note.* SE = standard error; corr. = adjusted for multiple testing using the Holm-method; Δ*R*^2^ = difference of adjusted *R*^2^ between consecutive models; MRNI-SF = Male Role Norms Inventory – Short Form; CMNI-30 = Conformity to Masculine Norms Inventory – 30; MC-SDS = Marlowe-Crowne Social Desirability Scale. Reference category is non-tertiary education, heterosexual, and single. Bold formatting for significant findings on an alpha-level of 5%. ^a^ standardized coefficient; ^raw^ unstandardized coefficient ^*^ *p* < .05; ^**^ *p* < .01; ^***^ p < .001 | | | | | | | |

| **Table S10** *Moderation Models with Aggression (ABPQ) as Outcome and Self-Compassion (SCS-SF) as Moderator* | | | | | | | |
| --- | --- | --- | --- | --- | --- | --- | --- |
| Predictor | | β^a^ (SE) | 95% CI | B^raw^ | *p* | *p* (corr.) | Δ*R*^2^ (%) |
| A) Endorsement of TMI as Predictor | | | | | | | |
| 1) | MRNI-SF | 4.68 (0.72) | [3.27, 6.10] | 0.50 | **<.001** | **<.001** |  |
|  | SCS-SF | -8.40 (0.71) | [-9.79, -7.00] | -0.55 | **<.001** | **<.001** |  |
|  | (MRNI-SF x SCS-SF) | -1.47 (0.66) | [-2.76, -0.17] | -0.01 | **.027** | **.027** |  |
|  |  |  |  |  |  |  | **34.2^***^** |
| 2) | MRNI-SF | 4.51 (0.67) | [3.19, 5.82] | 0.36 | **<.001** | **<.001** |  |
|  | SCS-SF | -6.09 (0.70) | [-7.48, -4.71] | -0.46 | **<.001** | **<.001** |  |
|  | (MRNI-SF x SCS-SF) | -0.79 (0.60) | [-1.98, 0.40] | -0.00 | .193 | .498 |  |
|  | Age | 1.00 (0.69) | [-0.35, 2.35] | 0.07 | .145 | .498 |  |
|  | Education | -4.33 (1.35) | [-6.99, -1.67] | -4.33 | **.001** | **.007** |  |
|  | Sexual orientation | 2.36 (1.53) | [-0.65, 5.38] | 2.36 | .124 | .498 |  |
|  | Relationship | -1.54 (1.33) | [-4.16, 1.09] | -1.54 | .250 | .498 |  |
|  | MC-SDS | -6.56 (0.68) | [-7.90, -5.23] | -2.96 | **<.001** | **<.001** |  |
|  |  |  |  |  |  |  | **12.4^***^** |
| 3) | MRNI-SF | 4.37 (0.66) | [3.06, 5.68] | 0.34 | **<.001** | **<.001** |  |
|  | SCS-SF | -6.11 (0.70) | [-7.48, -4.74] | -0.48 | **<.001** | **<.001** |  |
|  | (MRNI-SF x SCS-SF) | -0.73 (0.60) | [-1.91, 0.45] | -0.00 | .223 | .612 |  |
|  | Age | 0.87 (0.68) | [-0.47, 2.21] | 0.06 | .204 | .612 |  |
|  | Education | -4.38 (1.34) | [-7.01, -1.74] | -4.38 | **.001** | **.007** |  |
|  | Sexual orientation | 2.53 (1.52) | [-0.46, 5.53] | 2.53 | .097 | .389 |  |
|  | Relationship | -1.57 (1.32) | [-4.17, 1.03] | -1.57 | .236 | .612 |  |
|  | MC-SDS | -6.30 (0.68) | [-7.64, -4.97] | -2.84 | **<.001** | **<.001** |  |
|  | DV experienced | 4.89 (1.73) | [1.50, 8.29] | 4.89 | **.005** | **.024** |  |
|  |  |  |  |  |  |  | **0.9^**^** |
| B) Conformity to TMI as Predictor | | | | | | | |
| 1) | CMNI-30 | 5.95 (0.75) | [4.47, 7.42] | 0.55 | **<.001** | **<.001** |  |
|  | SCS-SF | -7.01 (0.74) | [-8.46, -5.55] | -0.44 | **<.001** | **<.001** |  |
|  | (CMNI-30 x SCS-SF) | -0.97 (0.63) | [-2.21, 0.27] | -0.01 | .126 | .126 |  |
|  |  |  |  |  |  |  | **36.4^***^** |
| 2) | CMNI-30 | 4.91 (0.73) | [3.48, 6.34] | 0.43 | **<.001** | **<.001** |  |
|  | SCS-SF | -5.43 (0.72) | [-6.85, -4.01] | -0.36 | **<.001** | **<.001** |  |
|  | (CMNI-30 x SCS-SF) | -0.70 (0.58) | [-1.84, 0.45] | -0.00 | .233 | .465 |  |
|  | Age | 2.13 (0.69) | [0.77, 3.50] | 0.14 | **.002** | **.009** |  |
|  | Education | -4.62 (1.35) | [-7.28, -1.95] | -4.62 | **<.001** | **.004** |  |
|  | Sexual orientation | 1.59 (1.52) | [-1.41, 4.59] | 1.59 | .297 | .465 |  |
|  | Relationship | -2.33 (1.33) | [-4.95, 0.28] | -2.33 | .081 | .242 |  |
|  | MC-SDS | -5.70 (0.70) | [-7.08, -4.32] | -2.57 | **<.001** | **<.001** |  |
|  |  |  |  |  |  |  | **9.9^***^** |
| 3) | CMNI-30 | 4.80 (0.72) | [3.38, 6.22] | 0.42 | **<.001** | **<.001** |  |
|  | SCS-SF | -5.46 (0.72) | [-6.86, -4.05] | -0.36 | **<.001** | **<.001** |  |
|  | (CMNI-30 x SCS-SF) | -0.68 (0.58) | [-1.81, 0.45] | -0.00 | .238 | .467 |  |
|  | Age | 1.96 (0.69) | [0.61, 3.31] | 0.13 | **.005** | **.018** |  |
|  | Education | -4.66 (1.34) | [-7.30, -2.02] | -4.66 | **<.001** | **.003** |  |
|  | Sexual orientation | 1.80 (1.51) | [-1.17, 4.77] | 1.80 | .233 | .467 |  |
|  | Relationship | -2.34 (1.32) | [-4.93, 0.25] | -2.34 | .077 | .230 |  |
|  | MC-SDS | -5.43 (0.70) | [-6.81, -4.05] | -2.45 | **<.001** | **<.001** |  |
|  | DV experienced | 5.30 (1.72) | [1.92, 8.69] | 5.30 | **.002** | **.011** |  |
|  |  |  |  |  |  |  | **1.1^***^** |
| *Note.* SE = standard error; OR = odds ratio; corr. = adjusted for multiple testing using the Holm-method; Δ*R*^2^ = difference of adjsuted R^2^ between consecutive models; MRNI-SF = Male Role Norms Inventory – Short Form; CMNI-30 = Conformity to Masculine Norms Inventory – 30; ERQ-suppress. = Emotion Regulation Questionnaire – Suppression subscale; MC-SDS = Marlowe-Crowne Social Desirability Scale. Bold formatting for statistically significant findings on an alpha-level of 5%. ^a^ standardized coefficient; ^raw^ unstandardized coefficient ^*^ *p* < .05; ^**^ *p* < .01; ^***^ p < .001 | | | | | | | |

| **Table S11** *Moderation Models with DV Perpetration as Outcome and Alexithymia (TAS-26) as Moderator* | | | | | | | | |
| --- | --- | --- | --- | --- | --- | --- | --- | --- |
| Predictor | | β^a^ (SE) | OR^a^ | 95% CI | OR^raw^ | *p* | *p* (corr.) | Δ*R*^2^ (%) |
| A) Endorsement of TMI as Predictor | | | | | | | | |
| 1) | MRNI-SF | 0.26 (0.13) | 1.30 | [1.00, 1.68] | 1.03 | **.046** | .139 |  |
|  | TAS-26 | 0.14 (0.14) | 1.14 | [0.86, 1.52] | 1.03 | .345 | .691 |  |
|  | (MRNI-SF x TAS-26) | -0.08 (0.12) | 0.92 | [0.73, 1.16] | 1.00 | .470 | .691 |  |
|  |  |  |  |  |  |  |  | 2.1 |
| 2) | MRNI-SF | 0.19 (0.15) | 1.21 | [0.90, 1.62] | 1.03 | .201 | .804 |  |
|  | TAS-26 | 0.17 (0.17) | 1.19 | [0.86, 1.65] | 1.04 | .305 | .914 |  |
|  | (MRNI-SF x TAS-26) | -0.12 (0.14) | 0.89 | [0.68, 1.16] | 1.00 | .384 | .914 |  |
|  | Age | 0.97 (0.19) | 2.64 | [1.82, 3.84] | 1.07 | **<.001** | **<.001** |  |
|  | Education | -0.97 (0.33) | 0.38 | [0.20, 0.73] | 0.38 | **.003** | **.021** |  |
|  | Sexual orientation | -0.91 (0.47) | 0.40 | [0.16, 1.01] | 0.40 | .052 | .260 |  |
|  | Relationship | 0.07 (0.32) | 1.07 | [0.58, 2.00] | 1.07 | .820 | .914 |  |
|  | MC-SDS | -0.49 (0.16) | 0.61 | [0.44, 0.84] | 0.80 | **.002** | **.017** |  |
|  |  |  |  |  |  |  |  | **17.5^***^** |
| 3) | MRNI-SF | 0.13 (0.16) | 1.14 | [0.83, 1.57] | 1.03 | .414 | 1 |  |
|  | TAS-26 | 0.21 (0.17) | 1.23 | [0.88, 1.73] | 1.05 | .232 | .928 |  |
|  | (MRNI-SF x TAS-26) | -0.13 (0.15) | 0.88 | [0.66, 1.17] | 1.00 | .370 | 1 |  |
|  | Age | 1.03 (0.21) | 2.79 | [1.86, 4.19] | 1.07 | **<.001** | **<.001** |  |
|  | Education | -1.16 (0.36) | 0.31 | [0.15, 0.64] | 0.31 | **.001** | **.010** |  |
|  | Sexual orientation | -0.89 (0.49) | 0.41 | [0.16, 1.08] | 0.41 | .071 | .356 |  |
|  | Relationship | 0.08 (0.34) | 1.09 | [0.56, 2.11] | 1.09 | .803 | 1 |  |
|  | MC-SDS | -0.37 (0.17) | 0.69 | [0.50, 0.97] | 0.85 | **.032** | .191 |  |
|  | DV experienced | 2.07 (0.35) | 7.91 | [4.00, 15.65] | 7.91 | **<.001** | **<.001** |  |
|  |  |  |  |  |  |  |  | **13.1^***^** |
| B) Conformity to TMI as Predictor | | | | | | | | |
| 1) | CMNI-30 | 0.21 (0.14) | 1.24 | [0.94, 1.63] | 1.07 | .132 | .298 |  |
|  | TAS-26 | 0.12 (0.14) | 1.12 | [0.84, 1.49] | 1.07 | .427 | .427 |  |
|  | (CMNI-30 x TAS-26) | -0.21 (0.13) | 0.81 | [0.63, 1.04] | 1.00 | .099 | .298 |  |
|  |  |  |  |  |  |  |  | 2.5 |
| 2) | CMNI-30 | 0.28 (0.17) | 1.33 | [0.95, 1.87] | 1.09 | .101 | .304 |  |
|  | TAS-26 | 0.14 (0.17) | 1.15 | [0.82, 1.60] | 1.10 | .416 | .831 |  |
|  | (CMNI-30 x TAS-26) | -0.30 (0.15) | 0.74 | [0.55, 1.00] | 1.00 | **.047** | .226 |  |
|  | Age | 1.06 (0.19) | 2.87 | [1.96, 4.21] | 1.07 | **<.001** | **<.001** |  |
|  | Education | -1.04 (0.34) | 0.35 | [0.18, 0.68] | 0.35 | **.002** | **.014** |  |
|  | Sexual orientation | -0.94 (0.47) | 0.39 | [0.15, 0.98] | 0.39 | **.045** | .226 |  |
|  | Relationship | 0.09 (0.32) | 1.09 | [0.59, 2.04] | 1.09 | .778 | .831 |  |
|  | MC-SDS | -0.43 (0.17) | 0.65 | [0.47, 0.91] | 0.82 | **.011** | .068 |  |
|  |  |  |  |  |  |  |  | **19.1^***^** |
| 3) | CMNI-30 | 0.30 (0.19) | 1.35 | [0.92, 1.98] | 1.11 | .121 | .407 |  |
|  | TAS-26 | 0.16 (0.17) | 1.18 | [0.83, 1.66] | 1.12 | .358 | .717 |  |
|  | (CMNI-30 x TAS-26) | -0.34 (0.16) | 0.71 | [0.52, 0.98] | 1.00 | **.040** | .240 |  |
|  | Age | 1.10 (0.21) | 3.01 | [1.99, 4.55] | 1.07 | **<.001** | **<.001** |  |
|  | Education | -1.25 (0.37) | 0.29 | [0.14, 0.59] | 0.29 | **<.001** | **.005** |  |
|  | Sexual orientation | -0.85 (0.49) | 0.43 | [0.16, 1.11] | 0.43 | .081 | .407 |  |
|  | Relationship | 0.09 (0.34) | 1.10 | [0.56, 2.14] | 1.10 | .782 | .782 |  |
|  | MC-SDS | -0.31 (0.18) | 0.73 | [0.52, 1.05] | 0.87 | .087 | .407 |  |
|  | DV experienced | 2.11 (0.35) | 8.27 | [4.13, 16.53] | 8.27 | **<.001** | **<.001** |  |
|  |  |  |  |  |  |  |  | **13.1^***^** |
| *Note.* SE = standard error; OR = odds ratio; corr. = adjusted for multiple testing using the Holm-method; Δ*R*^2^ = difference of Nagelkerke’s R^2^ between consecutive models; MRNI-SF = Male Role Norms Inventory – Short Form; CMNI-30 = Conformity to Masculine Norms Inventory – 30; ERQ-suppress. = Emotion Regulation Questionnaire – Suppression subscale; MC-SDS = Marlowe-Crowne Social Desirability Scale. Bold formatting for statistically significant findings on an alpha-level of 5%. ^a^ standardized coefficient; ^raw^ unstandardized coefficient ^*^ *p* < .05; ^**^ *p* < .01; ^***^ p < .001 | | | | | | | | |

| **Table S12** *Sensitivity Analyses for Moderation Models with DV Perpetration as Outcome and Expressive Suppression as Moderator* | | | | | | | | |
| --- | --- | --- | --- | --- | --- | --- | --- | --- |
| Predictor | | β^a^ (SE) | OR^a^ | 95% CI | OR^raw^ | *p* | *p* (corr.) | Δ*R*^2^ (%) |
| A) Total Sample | | | | | | | | |
| 1) | CMNI-30 | 0.32 (0.15) | 1.37 | [1.02, 1.85] | 1.10 | **.039** | .078 |  |
|  | ERQ-suppress. | -0.01 (0.15) | 0.99 | [0.74, 1.32] | 1.31 | .934 | .934 |  |
|  | (CMNI x suppress.) | -0.37 (0.14) | 0.69 | [0.53, 0.90] | 0.99 | **.006** | **.018** |  |
|  |  |  |  |  |  |  |  | **4.7^*^** |
| 2) | CMNI-30 | 0.33 (0.18) | 1.39 | [0.97, 1.99] | 1.11 | .075 | .226 |  |
|  | ERQ-suppress. | 0.08 (0.17) | 1.08 | [0.78, 1.50] | 1.38 | .642 | 1 |  |
|  | (CMNI x suppress.) | -0.42 (0.16) | 0.66 | [0.48, 0.89] | 0.99 | **.007** | **.047** |  |
|  | Age | 1.03 (0.19) | 2.82 | [1.92, 4.12] | 1.07 | **<.001** | **<.001** |  |
|  | Education | -0.91 (0.33) | 0.40 | [0.21, 0.78] | 0.40 | **.007** | **.047** |  |
|  | Sexual orientation | -0.99 (0.47) | 0.37 | [0.15, 0.95] | 0.37 | **.038** | .152 |  |
|  | Relationship | 0.05 (0.32) | 1.05 | [0.56, 1.94] | 1.05 | .884 | 1 |  |
|  | MC-SDS | -0.46 (0.18) | 0.63 | [0.45, 0.89] | 0.81 | **.009** | **.047** |  |
|  |  |  |  |  |  |  |  | **18.2^***^** |
| 3) | CMNI-30 | 0.34 (0.20) | 1.40 | [0.94, 2.09] | 1.10 | .099 | .308 |  |
|  | ERQ-suppress. | 0.09 (0.18) | 1.09 | [0.77, 1.55] | 1.32 | .627 | 1 |  |
|  | (CMNI x suppress.) | -0.36 (0.17) | 0.70 | [0.50, 0.97] | 0.99 | **.034** | .203 |  |
|  | Age | 1.09 (0.21) | 2.98 | [1.97, 4.50] | 1.07 | **<.001** | **<.001** |  |
|  | Education | -1.12 (0.36) | 0.33 | [0.16, 0.66] | 0.33 | **.002** | **.014** |  |
|  | Sexual orientation | -0.91 (0.49) | 0.40 | [0.15, 1.04] | 0.40 | .062 | .308 |  |
|  | Relationship | 0.05 (0.34) | 1.05 | [0.54, 2.03] | 1.05 | .885 | 1 |  |
|  | MC-SDS | -0.34 (0.18) | 0.71 | [0.49, 1.02] | 0.86 | .064 | .308 |  |
|  | DV experienced | 2.01 (0.35) | 7.45 | [3.74, 14.84] | 7.45 | **<.001** | **<.001** |  |
|  |  |  |  |  |  |  |  | **11.8^***^** |
| B) Heterosexual-Identified Men | | | | | | | | |
| 1) | CMNI-30 | 0.28 (0.16) | 1.32 | [0.96, 1.81] | 1.10 | .087 | .174 |  |
|  | ERQ-suppress. | 0.06 (0.16) | 1.06 | [0.77, 1.46] | 1.34 | .714 | .714 |  |
|  | (CMNI x suppress.) | -0.35 (0.15) | 0.70 | [0.52, 0.94] | 0.99 | **.017** | .051 |  |
|  |  |  |  |  |  |  |  | **4.7^*^** |
| 2) | CMNI-30 | 0.38 (0.19) | 1.46 | [1.00, 2.13] | 1.11 | **.048** | .144 |  |
|  | ERQ-suppress. | 0.13 (0.18) | 1.14 | [0.80, 1.62] | 1.38 | .458 | .915 |  |
|  | (CMNI x suppress.) | -0.37 (0.16) | 0.69 | [0.51, 0.94] | 0.99 | **.019** | .077 |  |
|  | Age | 1.07 (0.21) | 2.92 | [1.92, 4.42] | 1.07 | **<.001** | **<.001** |  |
|  | Education | -1.05 (0.37) | 0.35 | [0.17, 0.72] | 0.35 | **.004** | **.026** |  |
|  | Relationship | 0.20 (0.34) | 1.22 | [0.63, 2.38] | 1.22 | .551 | .915 |  |
|  | MC-SDS | -0.45 (0.19) | 0.64 | [0.44, 0.92] | 0.81 | **.015** | .074 |  |
|  |  |  |  |  |  |  |  | **17.8^***^** |
| 3) | CMNI-30 | 0.37 (0.21) | 1.45 | [0.96, 2.19] | 1.09 | .075 | .376 |  |
|  | ERQ-suppress. | 0.15 (0.19) | 1.16 | [0.80, 1.68] | 1.31 | .422 | .845 |  |
|  | (CMNI x suppress.) | -0.30 (0.17) | 0.74 | [0.53, 1.04] | 1.00 | .082 | .376 |  |
|  | Age | 1.08 (0.22) | 2.94 | [1.90, 4.57] | 1.07 | **<.001** | **<.001** |  |
|  | Education | -1.27 (0.40) | 0.28 | [0.13, 0.61] | 0.28 | **.001** | **.008** |  |
|  | Relationship | 0.24 (0.36) | 1.27 | [0.63, 2.58] | 1.27 | .507 | .845 |  |
|  | MC-SDS | -0.33 (0.20) | 0.72 | [0.49, 1.06] | 0.86 | .094 | .376 |  |
|  | DV experienced | 1.85 (0.38) | 6.36 | [3.03, 13.33] | 6.36 | **<.001** | **<.001** |  |
|  |  |  |  |  |  |  |  | **10.6^***^** |
| *Note.* SE = standard error; OR = odds ratio; corr. = adjusted for multiple testing using the Holm-method; Δ*R*^2^ = difference of Nagelkerke’s R^2^ between consecutive models; MRNI-SF = Male Role Norms Inventory – Short Form; CMNI-30 = Conformity to Masculine Norms Inventory – 30; ERQ-suppress. = Emotion Regulation Questionnaire – Suppression subscale; MC-SDS = Marlowe-Crowne Social Desirability Scale. Reference category is non-tertiary education, heterosexual, and single. Bold formatting for significant findings on an alpha-level of 5%. ^a^ standardized coefficient; ^raw^ unstandardized coefficient ^*^ *p* < .05; ^**^ *p* < .01; ^***^ p < .001 | | | | | | | | |
